# Graphical analysis of agent-based opinion formation models

**DOI:** 10.1371/journal.pone.0303204

**Published:** 2024-05-30

**Authors:** Carlos Andrés Devia, Giulia Giordano

**Affiliations:** 1 Delft Center for Systems and Control, Delft University of Technology, Delft, The Netherlands; 2 Department of Industrial Engineering, University of Trento, Trento, Italy; Roma Tre University: Universita degli Studi Roma Tre, ITALY

## Abstract

Agent-based models of opinion formation are becoming increasingly complex, because of their size and of the embedding of several individual psychological traits of the agents, aimed at realistically capturing the multifaceted aspects of social interaction. Therefore, the characterisation of the model properties mostly relies on simulation-based numerical approaches: more techniques are needed to analyse, contrast, and compare the properties of different models. We propose a novel graphical technique, which relies on the Agreement Plot to visualise the evolution of opinion distributions over time, that allows us to unveil behavioural patterns and capabilities of agent-based opinion formation models. Our proposed approach can be used to characterise the relation between global properties of the model evolution and the model features (initial opinion distributions, agent parameters, underlying digraphs), and is here showcased through its application to both seminal and recently proposed opinion formation models.

## Introduction

The interest of the scientific community in the mathematical modelling of opinion formation within a group of people has been growing in the last decades [1–15], leading to several seminal papers proposing and discussing new models [16–25].

Agent-based models describe how each individual within a group of interconnected agents (representing individuals in a population) evolves according to its internal dynamics and the information exchanged with other agents. A pioneering model of opinion formation that uses an agent-based approach is due to French [26], Harary [27, 28] and DeGroot [16]. In the French-DeGroot model, the new opinion of every agent is the weighted sum of its previous opinion and the opinion of its neighbours. This opinion update mechanism was inspired by the observation that, when presented with different perspectives within a discussion in a small group, individuals tend to agree with the general perceived opinion and thus converge to the weighted average of the initial opinions (consensus). Although accurate in some contexts, this model cannot be applied to large populations or long time scales: in those cases, opinion formation is driven by more mechanisms than sole averaging. Several additional mechanisms have been discovered by psychological and sociological research, and have been introduced into increasingly complex opinion formation models, which often build upon the original French-DeGroot model.

Individuals (or agents) not only use current opinions to form a new opinion, but are also influenced by prejudices, introduced in the Friedkin-Johnsen model [29, 30], which extends the French-DeGroot model by endowing each agent with an internal prejudice (which is often its initial opinion) that will influence its opinion evolution at every instant. The effect of this prejudice is determined by the agent *susceptibility*: a completely susceptible agent ignores its own prejudice and is fully open to influence from other agents, while a completely non-susceptible agent has a constant opinion equal to its own prejudice.

Some individuals are influenced only by agents that think like themselves and ignore agents with distant opinions. This trait, known as homophily, was introduced in the Bounded Confidence model, by Hegselmann and Krause [23], where each agent has a *confidence radius* and is only influenced by another agent when the difference between their opinions is smaller or equal to the confidence radius. This model is known for producing clusters, where agents in the same cluster have identical opinions, while the opinions to which different clusters converge have a difference that is larger or equal to the confidence radius of the agents.

Sometimes, trying to convince an individual to change its opinion results in that individual disagreeing even more than initially. This is known as the backfire effect, embedded in the BEBA (Backfire Effect and Biased Assimilation) model [31], where each agent has an intrinsic *entrenchment* parameter, representing how ‘convinced’ the agent is of its own opinion. When another agent tries to persuade a highly entrenched agent to change opinion, this attempt backfires and, as a result, the opinion difference between the two agents increases.

While the inclusion of mechanisms such as prejudices, homophily, and entrenchment (along with many others, such as *stubbornness* [32, 33], *biases* [34–36], *assimilation* [37, 38], *emotions* [39, 40]) makes the models more realistic, it also increases the difficulty of their formal analysis. The analytical study of opinion formation models has been very successful in some instances (see for instance [25, 41–50]), with a particular focus on achieving consensus (see e.g. [51–55]). However, the theoretical analysis of complex opinion formation models typically has to be restricted to special cases, and the complete model capabilities and properties are left to be investigated from a numerical perspective. This tendency of exploring the model behaviour, properties and capabilities through simulation-based analyses [56–59] has resulted in a number of simulation-based techniques that use distributional measures to characterise the relation between the initial conditions (initial opinion distribution) and the model outcomes (evolution of the opinion formation process, leading to a final opinion distribution at the end of a given horizon). In [60], a histogram-based sorting algorithm is proposed to classify opinion distributions, both observed in real life and generated by the evolution of opinion formation models, in order to uncover opinion transitions seen in actual populations and predicted by different agent-based models. In [61], a systematic approach is put forth to compute the probability that the final opinion distribution has desired qualitative features, when only incomplete information on the initial opinion distribution is available. In [38], the measures of *bias*, *diversity* and *fragmentation* are used to classify opinion distributions and relate model parameters with qualitative properties of the resulting opinions. Simulation-based techniques are thus central within frameworks used to classify, compare, and contrast different opinion formation models [8, 59, 60, 62].

This paper introduces the *Agreement Plot* as a novel analysis tool that relies on representing an opinion distribution *x* as a point in the Cartesian plane, with coordinates given by the average of the opinions, $\bar{x}$, and the average of the opinion absolute values, $\bar{\left|x\right|}$. Although it does not inform about the exact opinions, the location of the point provides relevant qualitative and quantitative information on the opinion distribution. Considering the case in which the opinion of each agent is a real number in the interval [−1, 1], a point located near the origin represents societies where most agents have a neutral or indifferent opinion, while a point with low $\bar{x}$ and high $\bar{\left|x\right|}$ corresponds to a polarised society, where agents have strong opinions that are evenly distributed between agreement and disagreement.

By associating entire opinion distributions (with an arbitrary number of agents) with a single point in the Cartesian plane, we can represent multiple opinion evolutions in a single plot. When distinct opinion evolutions differ only by the initial opinion distributions, or by the agent parameters, or by the underlying digraphs, the plot reveals how changes in these model features affect the opinion evolution and the final opinion distribution. Moreover, plotting the predicted final opinion distributions for a wide array of initial opinion distributions, agent parameters, and underlying digraphs provides a visual representation of the final opinion distributions that a given model is able to produce, which helps us understand how the model outcomes and behaviour are related to the model features, and can also reveal intrinsic properties of the model.

Our proposed technique can be applied to any agent-based opinion formation model in which the opinion of an agent belongs to a bounded interval in the real line. As a case-study, we consider the Friedkin-Johnsen model [29, 30] and the recently proposed Classification-based model [63] (in the Main Paper) as well as the Bounded Confidence model [23] and the recently proposed Backfire Effect and Biased Assimilation model [31] (in the S1 File). A detailed description of all these models is provided in the S1 File.

## Methods

We introduce the concept of **Agreement Plot**, based on which we define six different types of plots: Agent Parameter Time Evolution (**APTE**), Underlying Digraph Time Evolution (**UDTE**), Initial Opinion Time Evolution (**IOTE**), Agent Parameter Steady State (**APSS**), Underlying Digraph Steady State (**UDSS**), and Initial Opinion Steady State (**IOSS**). Collectively, these six plots can be used to analyse different opinion formation models and identify their behavioural patterns and intrinsic characteristics. The different features of the six plot types are summarised in Table 1.

**Table 1:** Overview of the six considered Agreement Plot types to analyse agent-based opinion formation models.

Plot type	What is visualised in the Agreement Plot	Initial opinion distribution	Agent parameters	Underlying digraph	Example
**APTE**	Curves showing the time evolution of the opinion distributions for a given model dynamics.	Constant	Changed	Constant	Fig 2A
**UDTE**		Constant	Constant	Changed	Fig 2B
**IOTE**		Changed	Constant	Constant	Fig 2C
**APSS**	Steady-state opinion distributions resulting from the long-term evolution of a given model dynamics.	Constant	Changed	Constant	Fig 4A
**UDSS**		Constant	Constant	Changed	Fig 4B
**IOSS**		Changed	Constant	Constant	Fig 4D

Throughout the Methods section, our proposed technique is exemplified by considering the Friedkin-Johnsen model [29, 30] to generate our plots.

### Agreement Plot

We consider a population of *N* individuals, or agents, and we denote by *x*_*i*_ ∈ [−1, 1] the opinion of agent *i*. Given an *opinion distribution* $x={\left(x_{i}\right)}_{i=1}^{N}\in {\left[-1,1\right]}^{N}$ (namely, the vector of all opinions), the point *π*(*x*) defined as

$$\begin{array}{c} \pi \left(x\right)=(\bar{\left|x\right|},\bar{x}),\;\text{where}\;\bar{\left|x\right|}=\frac{1}{N}\sum_{i=1}^{N}\left|x_{i}\right|\;\text{and}\;\bar{x}=\frac{1}{N}\sum_{i=1}^{N}x_{i}, \end{array}$$ (1)

represents how strongly the population cares about ($\bar{\left|x\right|}$) and agrees with ($\bar{x}$) a statement. Since *x*_*i*_ ∈ [−1, 1] for all *i*, the point *π*(*x*) in the Cartesian plane is contained in the triangle with vertices in the points (0, 0), (1, −1) and (1, 1), regardless of the number *N* of agents. We call **Agreement Plot** the visualisation of *π*(*x*) for one or more opinion distributions in the Cartesian plane.

The location of the point *π*(*x*) provides useful information about the opinion distribution. For instance, if $\bar{\left|x\right|}\approx 1$, the population cares a lot about the statement: most of the opinions are very strong, close to either −1 or 1. When $\bar{x}\approx 1$ or $\bar{x}\approx -1$, almost every agent must have a similar opinion, which is either ‘strongly agree’ or ‘strongly disagree’. Hence, a point located near (1, 1), or near (1, −1), represents a population where consensus has been practically reached towards strong agreement, or strong disagreement, with the statement. Conversely, if *π*(*x*) is in the neighbourhood of the corner (0, 0), then the average opinion is neutral ($\bar{x}\approx 0$) and most agents are indifferent about the statement ($\bar{\left|x\right|}\approx 0$). If *π*(*x*) ≈ (1, 0), then the population is polarised: the agents care a lot about the statement ($\bar{\left|x\right|}\approx 1$), but the average opinion is neutral ($\bar{x}\approx 0$), therefore two opposite groups must exist, with almost equal number of agents, associated with strong agreement and with strong disagreement. When *π*(*x*) is located near the lines $\bar{x}=\pm \bar{\left|x\right|}$, almost the whole population either agrees or disagrees to some degree. On the other hand, if *π*(*x*) is located near the line $\bar{x}=0$, there is an almost equal level of agreement and disagreement.

Here, we propose new types of model analysis that leverage this representation of an opinion distribution as a single point in the Cartesian plane, with a simple and intuitive interpretation. In particular, we build upon the Agreement Plot to analyse:

- the **time evolution** of the opinion distribution: a curve in the Cartesian plane, formed by points that represent the opinion distribution at different time instants, is generated over a certain time horizon (in the time evolution figures shown in the paper, we consider 50 time steps, unless stated otherwise) by the considered model with assigned parameters starting from a given initial condition;
- the opinion distribution at **steady state**: a single point in the Cartesian plane represents the final opinion distribution generated by the considered model with assigned parameters starting from a given initial condition (in the steady-state figures shown in the paper, we consider 1000 time steps).

In both types of analysis, the plot points (and curves) have a colour coding, which—unless otherwise specified—is model-dependent: the colour represents the average agent parameters of the considered population. For the Friedkin-Johnsen model, the agent parameter is susceptibility and λ_*i*_ ∈ [0, 1] denotes the susceptibility of agent *i*: the colour encodes the average population susceptibility $\bar{\lambda }$, with magenta associated with smaller $\bar{\lambda }$ and teal associated with larger $\bar{\lambda }$ (see also the agent parameter histograms in Fig 5). Conversely, for the Classification-based model, each agent has three parameters: the weights of the conformist trait *α*_*i*_ ∈ [0, 1], radical trait *β*_*i*_ ∈ [0, 1] and stubborn trait *γ*_*i*_ ∈ [0, 1], for agent *i*, which add up to 1. The RGB colour obtained by combining a fraction $\bar{\alpha }$ of blue, a fraction $\bar{\beta }$ of red and a fraction $\bar{\gamma }$ of green encodes the average conformist ($\bar{\alpha }$), radical ($\bar{\beta }$) and stubborn ($\bar{\gamma }$) trait weights (see also the agent parameter ternary diagram in Fig 12; the interpretation of ternary diagrams is discussed in the S1 File).

### Agreement Plot—Time evolution

The Agreement Plot can show one or more parametric curves (with the parameter being time) corresponding to the temporal evolution of the opinion distributions for one or more different populations.

**Fig 1:**
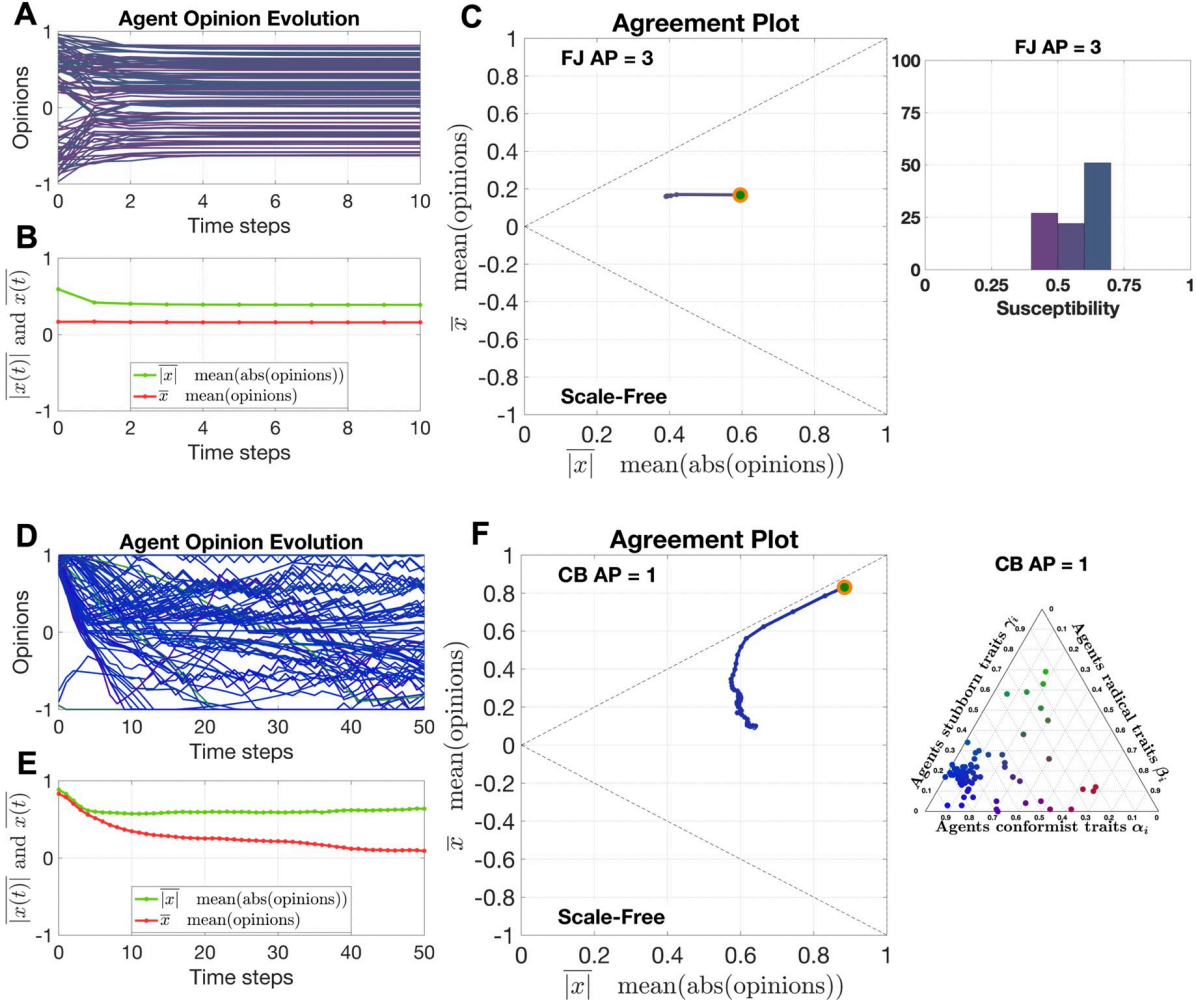
A single parametric curve in the Agreement Plot can capture the opinion evolution *x*(*t*) for a whole population (in this case, of *N* = 100 agents). In panels A-B-C, the opinion formation is described by the Friedkin-Johnsen model [29, 30], where the agents start from random initial conditions, have the parameters shown in the histogram ‘FJ AP = 3’ in Fig 5 (the histogram is also shown to the right of panel C), and evolve over a digraph with Scale-Free topology for 10 time steps. In panels D-E-F, the opinion formation is described by the Classification-based model [63], where the agents start from random initial conditions, have the parameters shown in the ternary diagram ‘CB AP = 1’ in Fig 12 (also shown to the right of panel F), and evolve over a digraph with Scale-Free topology for 50 time steps. Panels A/D: time evolution of the individual opinions *x*_*i*_(*t*), *i* = 1, …, *N*, for all the agents in the population; the colour of each curve encodes the parameter values associated with the individual agent. Panels B/D: time evolution of the average of the opinion absolute values, $\bar{\left|x(t)\right|}$, in green, and of the average of the opinions, $\bar{x(t)}$, in red, for the whole population. Panels C/F: corresponding parametric curve *π*(*x*(*t*)) in the Agreement Plot; the curve colour encodes the average susceptibility for the Friedkin-Johnsen model, and the average radicalism-stubborness-conformism weights (RGB colour) for the Classification-based model, while an orange circle denotes the initial opinion distribution.

Fig 1 shows two examples, considering the Friedkin-Johnsen model [29, 30] and the Classification-based model [63], of how the time evolution of the opinions within a population can be represented by a single parametric curve in the Agreement Plot. Fig 1A and 1D show the time evolution of the individual agent opinions *x*_*i*_(*t*), for all *i*. Fig 1B and 1E show the time evolution of $\bar{\left|x(t)\right|}$ (green) and of $\bar{x(t)}$ (red) for the whole population. In Fig 1C and 1F, the resulting parametric curve $\pi \left(x\left(t\right)\right)=(\bar{\left|x(t)\right|},\bar{x(t)})$ is shown in the Agreement Plot. In particular for the case of the Classification-based model, it is hard to gain insight by looking at the time evolution of the individual opinions: collective behaviours, hidden in the complex evolution of all the individual agents, are better visualised by the Agreement Plot.

**Fig 2:**
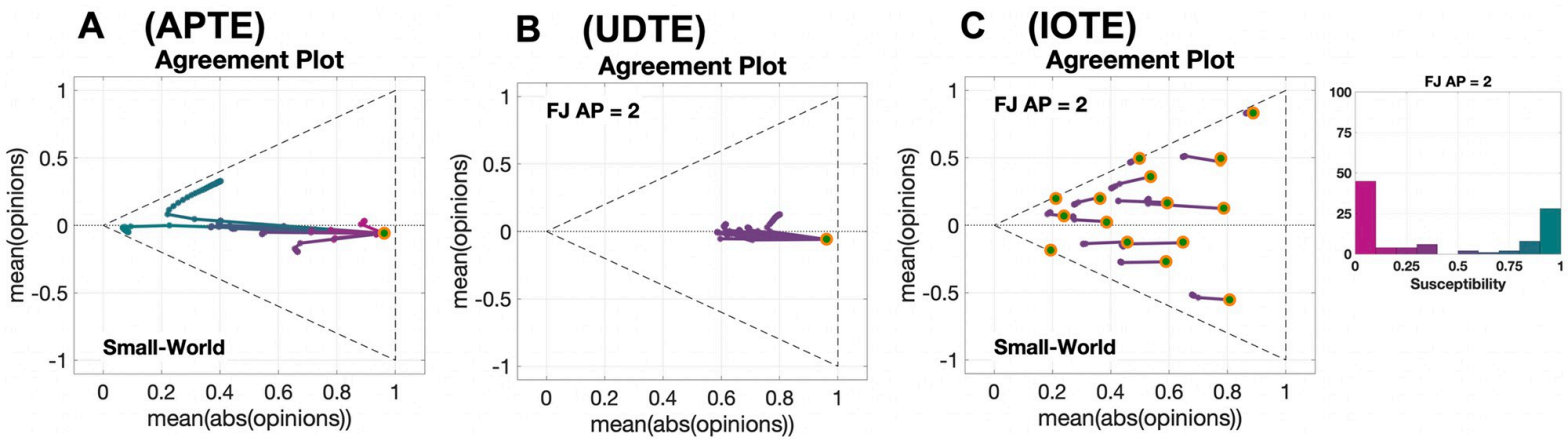
Agreement Plots that visualise different opinion evolution behaviours produced by the Friedkin-Johnsen model. Each plot shows several parametric curves (where the parameter is time) that visualise different opinion evolutions for populations that differ only in the agent parameters (A), in the underlying digraph (B), or in the initial opinion distribution (C). The line colour encodes the average susceptibility of the agents (less susceptible, magenta; more susceptible, teal). The initial opinion distributions are denoted by orange circles. The underlying digraph in Panels A and C is a Small-World digraph (see Table 2 for graph metrics). The agent parameters for Panels B and C are shown in the histogram on the right (see also the histogram ‘FJ AP = 2’ in Fig 5). All the simulations were performed for 50 time steps and 100 agents. Panel A: Agent Parameter Time Evolution (**APTE**) plot with 15 parametric curves, corresponding to 15 sets of agent parameters; the initial opinions and underlying digraph remained constant and the agent parameters changed. Panel B: Underlying Digraph Time Evolution (**UDTE**) plot with 45 parametric curves, corresponding to 45 different digraphs; the initial opinions and agent parameters remained constant and the underlying digraph changed. Panel C: Initial Opinion Time Evolution (**IOTE**) plot with 15 parametric curves, corresponding to 15 initial opinion distributions; the agent parameters and underlying digraph remained constant and the initial opinions changed.

Visualising in a single Agreement Plot the opinion evolutions over time for populations that differ in only one aspect, such as different agent parameters, underlying digraph or different initial opinion distribution, shows the overall effect of the considered change on the opinion formation process, as illustrated in Fig 2 for the Friedkin-Johnsen model.

The Agent Parameter Time Evolution (**APTE**) plot in Fig 2A shows 15 parametric curves corresponding to the opinion evolution with 15 different choices of the agent parameters, emphasised by the different colour, starting from the same initial opinion distribution (orange circle) and with the same underlying digraph (Small-World). Magenta curves tend to be shorter than teal curves, because the agents are less susceptible on average and therefore the opinions change less; teal curves correspond to populations with high average susceptibility, which leads to bigger opinion changes.

**Fig 3:**
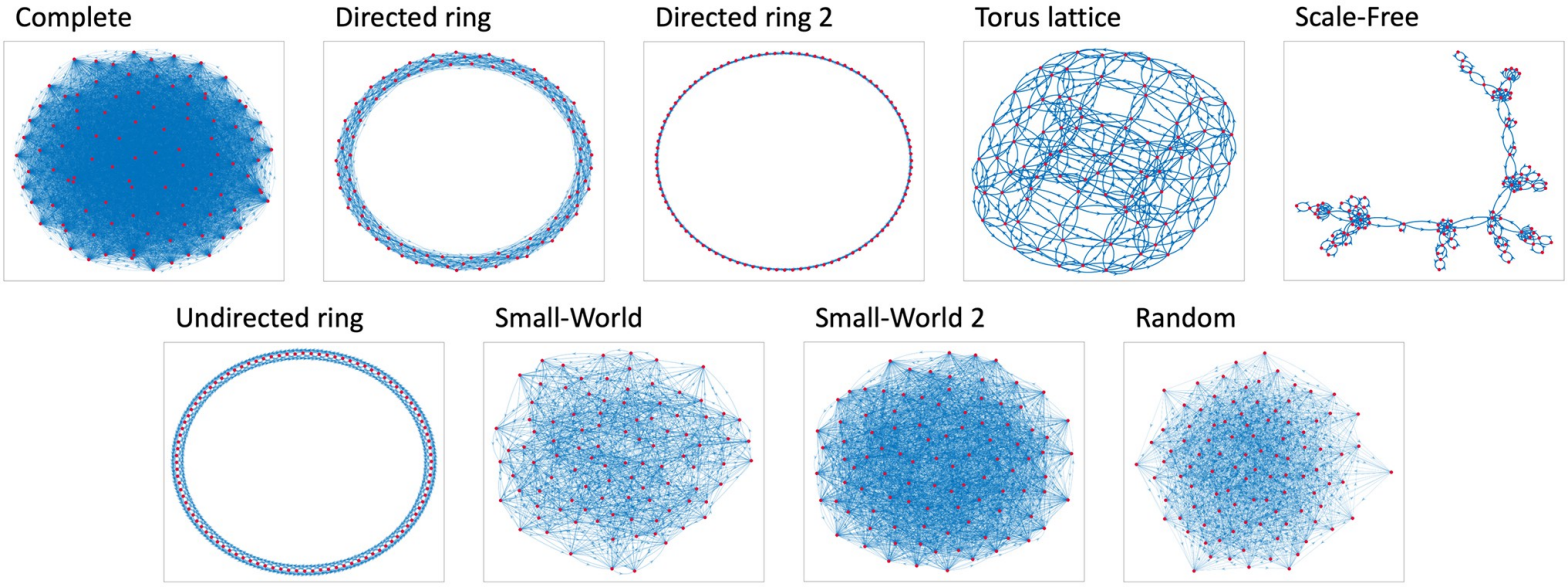
The 9 digraph topologies used to create the 45 different digraphs for UDTE and UDSS plots. Based on each of the 9 topologies, 5 digraphs were created. For the FJ model, 5 different choices of the weights were associated with the edges, so as to guarantee the corresponding adjacency matrix to be row stochastic. For the CB model, 5 different choices of the signs were associated with the edges. Some graph metrics for these topologies can be seen in Table 2.

The Underlying Digraph Time Evolution (**UDTE**) plot in Fig 2B shows 45 parametric curves corresponding to the opinion evolution evolving over 45 different underlying digraphs (the digraph topologies can be seen in Fig 3), with the same initial opinion (the orange dot) and agent parameters (shown in the histogram ‘FJ AP = 2’ in Fig 5, and also to the right of Fig 2). Regardless of the underlying digraph, there is a trend to move towards the left, as with **APTE** plots.

The Initial Opinion Time Evolution (**IOTE**) plot in Fig 2C shows 15 parametric curves corresponding to the opinion evolution starting from 15 different initial opinion distributions (orange circles), with the same agent parameters (shown in the histogram ‘FJ AP = 2’ in Fig 5, and to the right of Fig 2) and underlying digraph (Small-World). Independent of the initial opinion distribution, there is a tendency to move towards the left, but opinion distributions close to the lines $\bar{x}=\pm \bar{\left|x\right|}$ tend to change very little.

Do the behaviours emerging from Fig 2 depend on the chosen initial opinion distributions, agent parameters, and underlying digraphs? For instance, how would Fig 2 change if we changed the digraph topology from Small-World to Complete, or Scale-Free? To answer such questions, we can consider the same type of Agreement Plots generated for a variety of different initial opinions, agent parameters, and underlying digraphs, as shown in Fig 6 (for **APTE**), Fig 7 (for **UDTE**), and Fig 8 (for **IOTE**).

### Agreement Plot—Steady state

Assume that we are not interested in the opinion trajectory, but in the opinions at the start and at the end of a given time interval. In particular, the time interval could be such that the opinions are allowed to reach a ‘steady state’ with respect to the Agreement Plot: this means that the representation of the opinion distribution in the Agreement Plot remains the same, even though the actual opinion distribution and the individual opinions may well change. We refer to this concept of ‘steady state’ in the following. Then, in this scenario, we can produce an Agreement Plot that only shows the initial and final opinion distributions. Showing the opinion distribution at the steady state for several different choices of the agent parameters and of the initial opinion distribution (while keeping the other model features constant) can highlight what kind of opinion distributions the model can produce (starting from a given initial condition) and also how the model transforms the opinions in the long term. With respect to time evolution plots, for steady-state plots the simulation horizon should be increased (in our figures, from 50 to 1000 time steps), so that the models are given enough time to evolve and show also the opinion changes caused by slow dynamics.

**Fig 4:**
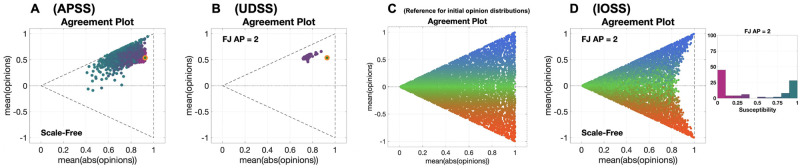
Agreement Plots that visualise different predicted opinion distributions produced by the Friedkin-Johnsen model for varying agent parameters (A), underlying digraphs (B), or initial opinion distributions (C). The plots show several predicted opinion distributions for different initial conditions. The point colour encodes the average susceptibility of the agents (less susceptible, magenta; more susceptible, teal). The initial opinion distributions are denoted by orange circles. For panels A and B, the orange dot represents the initial opinion distribution, and the other dots correspond to different and independent opinion predictions after 1000 time steps. The initial opinion for any point in Panel D is the point of the same colour in Panel C. The underlying digraph in Panels A and D was a Scale-Free digraph (see Table 2 for graph metrics). The agent parameters for Panels B and D are shown in the histogram to the right (and also in the histogram ‘FJ AP = 2’ in Fig 5). All the simulations were performed for 1000 time steps and 100 agents. Panel A: Agent Parameter Steady State (**APSS**) plot with 3528 predicted final opinion distributions, starting from the same initial opinion distribution (orange circle), corresponding to 3528 sets of agent parameters; the initial opinions and underlying digraph remained constant and the agent parameters were changed. Panel B: Underlying Digraph Steady State (**UDSS**) plot with 45 predicted opinion distributions, corresponding to 45 different digraphs (see Fig 3); the initial opinions and agent parameters remained constant and the underlying digraph changed. Panel C: reference plot for the Initial Opinion Steady State (**IOSS**) plots, showing the location in the Agreement Plot of the 5314 initial opinion distributions used to generate all the **IOSS** plots shown in the paper. Panel D: **IOSS** plot with 5314 predicted final opinion distributions, corresponding to 5314 initial opinion distributions; the agent parameters and underlying digraph remained constant and the initial opinions changed.

Agent Parameter Steady State (**APSS**) plots show the different final opinion distributions that can be achieved, always starting from the same initial opinion distribution, with different agent parameters. Fig 4A shows an example of **APSS** plot for the Friedkin-Johnsen model: 3528 final opinion distributions are computed, associated with the same initial opinion distribution (orange circle) and underlying digraph (Scale-Free), but with different agent parameters (as emphasised by the different colours of the points). The emerging behaviour is consistent with the **APTE** plot in Fig 2A: for instance, magenta points are closer to the orange circle than teal points. Very few points can be found below the line $\bar{x}=0$ or to the right of the initial opinions: this suggests that, for an opinion evolution driven by the Friedkin-Johnsen model, the sign of $\bar{x}$ is more likely to remain constant, while $\bar{\left|x\right|}$ is unlikely to increase.

Underlying Digraph Steady State (**UDSS**) plots present the final opinion distributions resulting from different underlying digraphs, for a fixed choice of the agent parameters and initial opinions. Fig 4B shows an example of **UDSS** plot for the Friedkin-Johnsen model: 45 final opinion distributions are computed, associated with the same initial opinion distribution (orange circle) and agent parameters (‘FJ AP = 2’, shown to the right of Figs 4 and at 5), but with different underlying digraphs (see Fig 3). The resulting behaviour is consistent with the **UDTE** plot in Fig 2B: the final opinion distributions have a lower absolute value mean, while the opinion mean remains approximately constant, meaning that opinions tend to converge to the weighted initial opinion mean, as expected in the FJ model.

**Fig 5:**
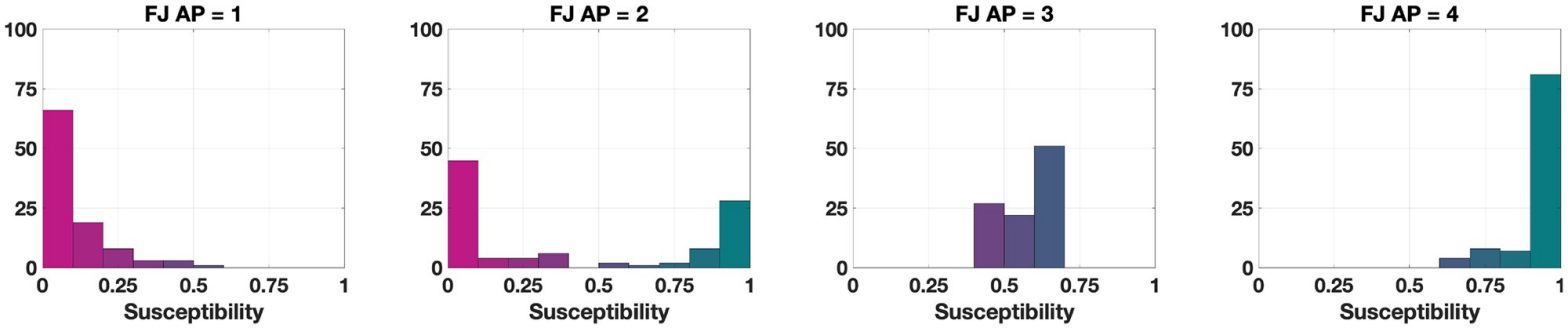
Histograms of different agent parameter choices for the Friedkin-Johnsen model with *N* = 100 agents. The height of each bin indicates the number of agents whose susceptibility parameter lies within the considered interval.

Initial Opinion Steady State (**IOSS**) plots, which visualise the final opinion distributions obtained from different initial opinions, for a given choice of the agent parameters and underlying digraph, need to be built differently. We start with the reference plot in Fig 4C, where each point corresponds to a different initial opinion distribution and has a unique colour. Then, the **IOSS** plot shows the final opinion distributions obtained by evolving the model, with the same agent parameters and underlying digraph, for each of the considered initial opinion distributions; an example, for the Friedkin-Johnsen model, is shown in Fig 4D. Two points with the same colour, one from the reference plot in Fig 4C and the other from the **IOSS** plot (e.g. Fig 4D), represent the initial opinion distribution and the final opinion distribution for the same simulated evolution.

Fig 4D shows how the 5314 initial opinions shown in Fig 4C transform according to the Friedkin-Johnsen model, always with the same agent parameters (shown in the histogram ‘FJ AP = 2’ in Fig 5, and to the right of Fig 4) and underlying digraph (Scale-Free). The contraction of the initial opinions towards the left, seen in Fig 4D, is more pronounced when $\bar{x}$ is closer to zero: strongly polarised opinions are transformed into less polarised opinions, consistently with the averaging tendency induced by the susceptibility trait in the Friedkin-Johnsen model. Also, the average opinion remains almost unchanged, as revealed by the fact that the colour gradient in Fig 4C is preserved in Fig 4D.

It is important to stress once again that, for the **APSS**, **UDSS**, and **IOSS** plots, *steady state* is defined *with respect to the Agreement Plot*. A population has achieved its steady state with respect to the Agreement Plot when the point *π*, as defined in Eq (1), does no longer change. However, this does not mean that the individual opinions, or the actual opinion distribution, remain the same; it only means that, regardless of whether they change or not, this is not reflected in the Agreement Plot, because the corresponding point in the Agreement Plot remains the same. We can assume that after 1000 time steps the populations have achieved their steady state with respect to the Agreement Plot, based on what is shown in the Temporal Evolution plots. Indeed, by looking at all the parametric curves, it is clear that even after 50 time steps most populations have reached their steady state, and after 1000 time steps all populations have.

Furthermore, it is worth highlighting that the **APSS**, **UDSS**, and **IOSS** plots are useful even if the populations have not achieved steady state. For instance, **APSS**, **UDSS**, and **IOSS** created at 5, 10, 50, and 100 time steps can be used to assess how the final opinions ‘expand’ and ‘cover’ the Agreement Plot. This can be done because the main property of the **APSS**, **UDSS**, and **IOSS** plots is that (differently from the **APTE**, **UDTE**, and **IOTE** plots, which capture time evolution) they represent the state of many different social systems (characterised by different parameters, or underlying digraphs, or initial opinions) after a given simulation time.

Arranging together multiple plots of the same type, as in Fig 9 (**APSS**), Fig 10 (**UDSS**) and Fig 11 (**IOSS**), allows us to determine whether the observed behaviour is intrinsic for the model, or whether (and how) it depends on the chosen initial conditions, agent parameters and underlying digraph.

## Results

To demonstrate our proposed methodology, we apply the new graphical analysis technique to the classical Friedkin-Johnsen model [29, 30] and the recently proposed Classification-based model [63]; the Bounded Confidence model [23] and the recently proposed Backfire Effect and Biased Assimilation model [31] are also analysed, in the S1 File. All the considered models are deterministic. We consider four possible topologies for the underlying digraphs over which the model evolves: Directed Ring, Scale-Free, Small-World, Complete. Relevant metrics for our considered topologies are shown in Table 2.

**Table 2:** Relevant metrics for the digraph topologies considered in the Agreement Plot analysis: Average path Length, APL; Diameter, D; Average clustering, $\bar{CC}$; Clustering variance, *σ*(*CC*); Number of edges, #E; Average out-degree, $\bar{\delta^{out}}$; Out-degree variance, *σ*(*δ*^*out*^); Average in-degree, $\bar{\delta^{in}}$; In-degree variance, *σ*(*δ*^*in*^); Bidirectional Coefficient, Bc. A description of how these graph metrics are computed is provided in the S1 File.

Topology	APL	Di	$\bar{CC}$	*σ*(*CC*)	#E	$\bar{\delta^{out}}$	*σ*(*δ*^*out*^)	$\bar{\delta^{in}}$	*σ*(*δ*^*in*^)	Bc
Complete graph	1	1	1	0	100	1	0.56346	1	1.4081e-31	1
Directed ring	5.4545	10	0.5	0	100	1	0.45168	1	2.2909e-32	0
Directed ring 2	10.404	20	0.5	0	100	1	0.44644	1	1.1952e-32	0
Undirected ring	5.4545	10	0.66667	4.9802e-32	100	1	0.57148	1	2.0792e-32	1
Small-World	2.0365	3	0.17538	0.0020628	100	1	0.47947	1	2.6644e-32	0.69357
Random network	1.8129	3	0.20122	0.00057076	100	1	0.56334	1	3.4737e-32	0.2243
Torus lattice	5.0505	10	0	0	100	1	0.37493	1	1.4318e-32	1
Scale-Free	5.6893	14	0.67	0.22333	100	1	0.94087	1	6.8478e-33	1
Small-World 2	1.6778	2	0.32651	0.00036845	100	1	0.4836	1	4.6565e-32	0.65038

### Analysis of the Friedkin-Johnsen model

We adopt our method to assess the opinion evolution generated by the Friedkin-Johnsen model [29, 30], for several different choices of the agent parameters (susceptibility, see Fig 5 for some examples), four different underlying digraph topologies (whose metrics are reported in Table 2) and several different initial conditions.

**Fig 6:**
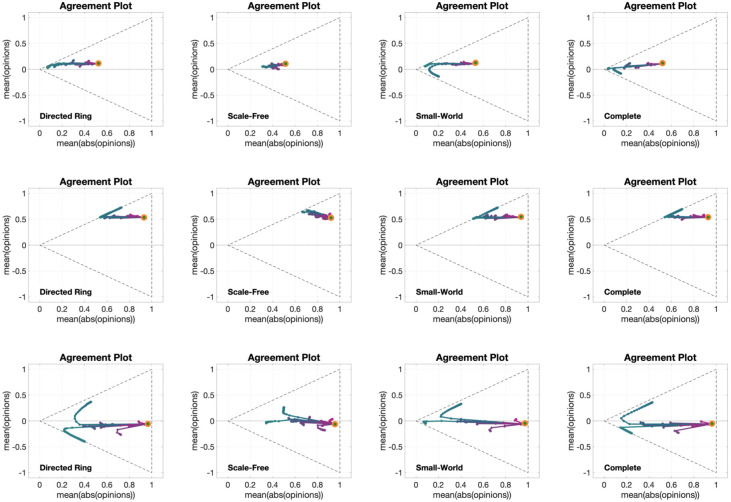
APTE plots for the Friedkin-Johnsen model. Each of the 12 plots includes 15 curves associated with different choices of the agent parameters, all with the same initial opinion distribution and underlying digraph. Plots in the same row have the same initial opinion distribution (orange circle). Plots in the same column have the same underlying digraph (from left to right, Directed Ring, Scale-Free, Small-World, Complete). All the simulations were performed for 50 time steps and 100 agents.

Fig 6 shows 12 different **APTE** plots, for three different choices of the initial opinion distribution (orange circles) and four different underlying digraphs. Although the overall qualitative behaviour of the plots in Fig 6 is the same as in Fig 2A, the graph topology appears to have an important effect on the opinion evolution: for all the considered initial opinions, the opinions change less when the model evolves over the Scale-Free digraph, whereas no significant difference can be noticed among the evolutions with the other three digraphs, in particular for the second row. A possible explanation for this phenomenon is that the large diameter and clustering variance of the Scale-Free digraph, combined, lead to a graph that has a few central, highly influential vertices connecting otherwise disconnected subgraphs: hence, the opinions need more time to spread, and thus they change less within the same time horizon. The other three graph topologies are very different among them; still, simulation results look very similar, in particular for the Directed Ring and Complete digraphs (first and fourth columns), even though for some of the metrics in Table 2 (including average path length and diameter) one has the highest value, while the other has the lowest value. This suggests that, for the Friedkin-Johnsen model, the opinion evolution is independent of these graph metrics.

**Fig 7:**
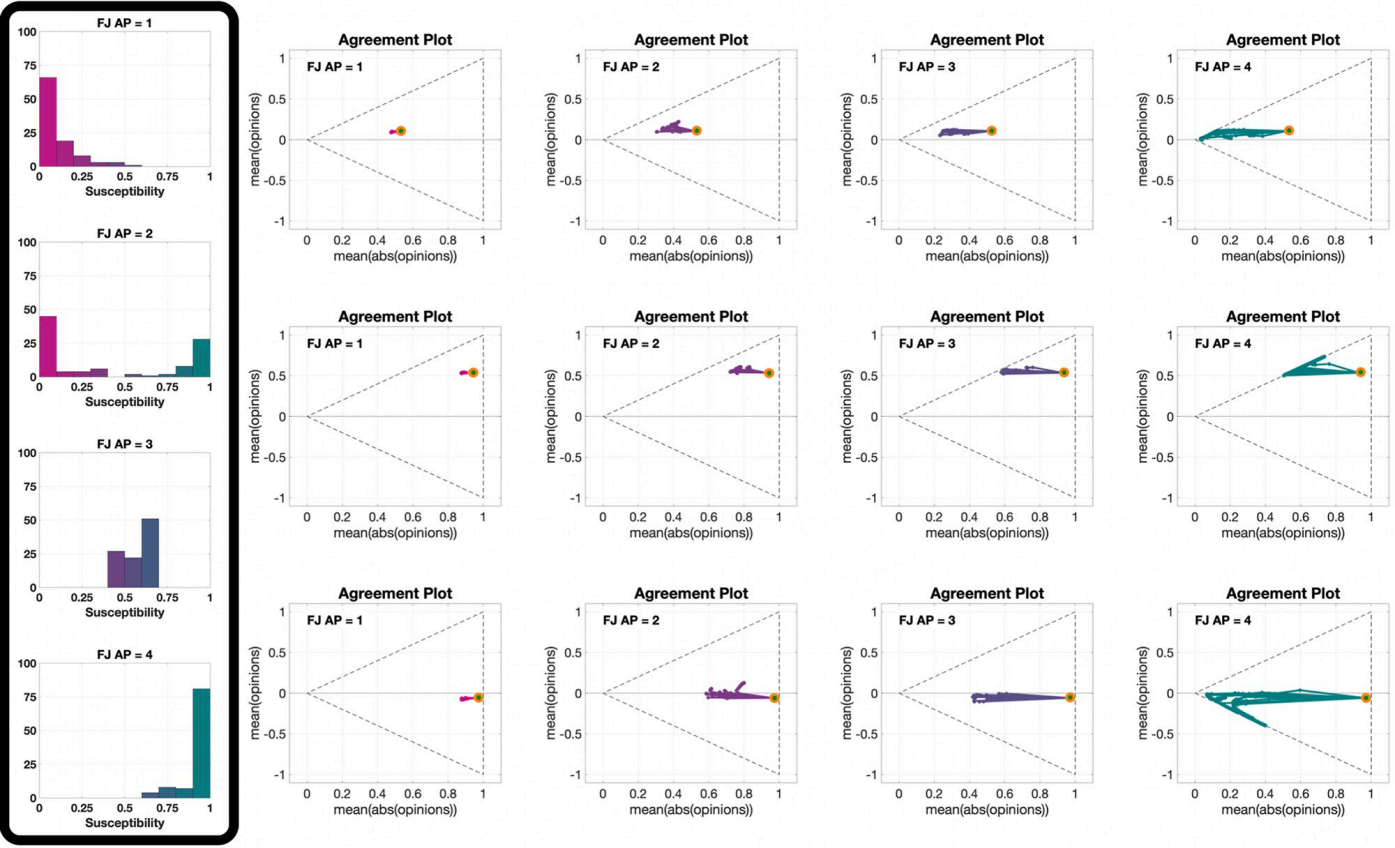
UDTE plots for the Friedkin-Johnsen model. Each of the 12 plots includes 45 curves associated with different choices of underlying digraphs, all with the same initial opinion distributions and agent parameters. Plots in the same row have the same initial opinion distribution (orange circle). Plots in the same column have the same agent parameters (the histograms of these agent parameters can be seen in the box to the left of the figure and in Fig 5). All the simulations were performed for 50 time steps and 100 agents.

Fig 7 presents 12 **UDTE** plots, for four different choices of the agent parameters and three different initial opinions. The fact that multiple lines are close together indicates that all the evolutions have more or less the same behaviour for all different digraphs we considered (shown in Fig 3). This could be surprising, taking into account that Fig 6 showed examples where the digraph has a significant effect on the opinion evolution. This apparent contradiction (of the digraphs not having a significant effect on the opinion evolution in one figure, and then having a significant effect on another figure) can be explained be noting that the digraph effect observed in Fig 6 was on the speed of opinion change, but not the direction of the curves, and that the plots in Fig 7 are showing exactly the same directions for parametric curves of different length.

**Fig 8:**
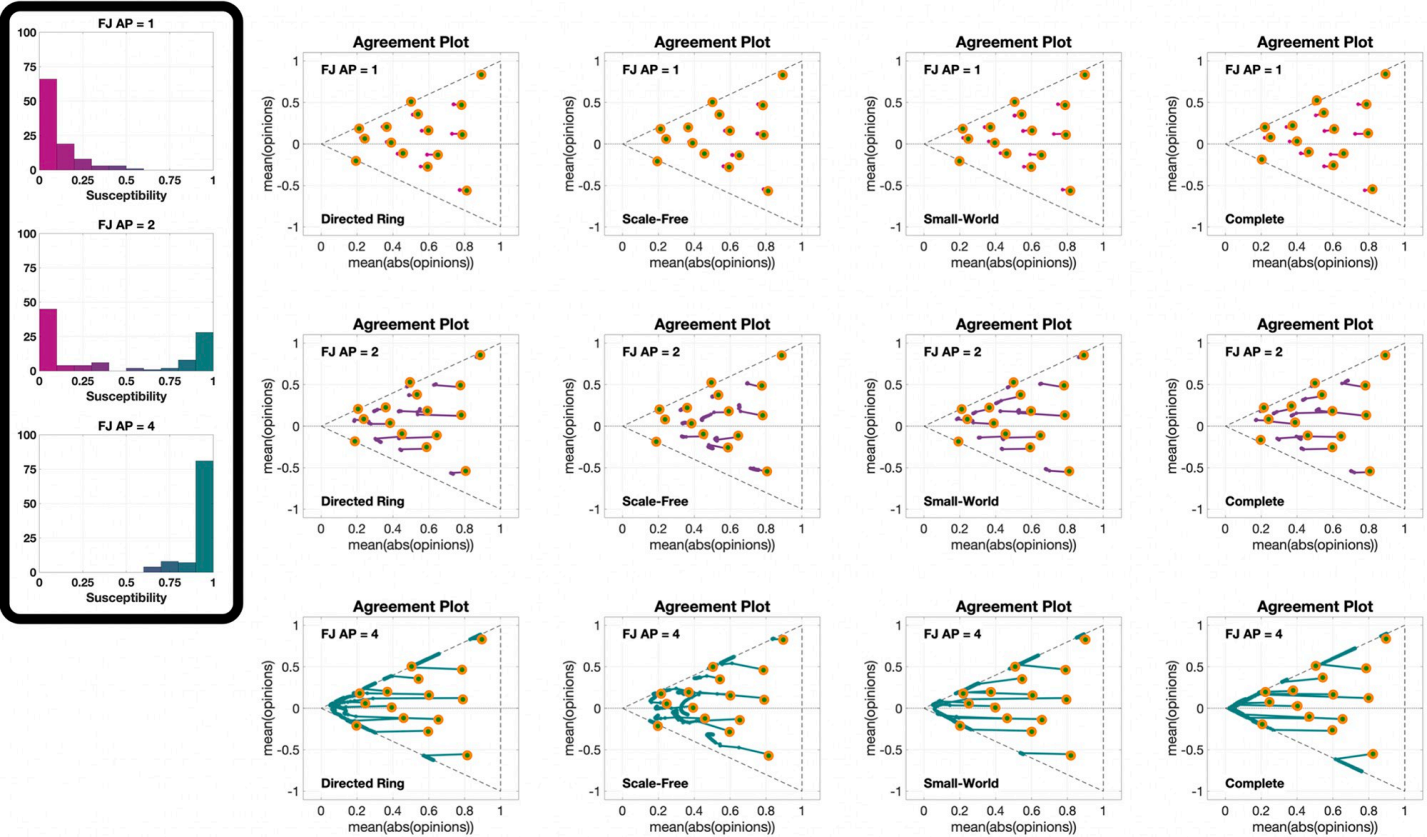
IOTE plots for the Friedkin-Johnsen model. Each of the 12 plots includes 15 curves associated with different choices of the initial opinion distributions, all with the same agent parameters and underlying digraph. Plots in the same row have the same agent parameters (the histograms of these agent parameters can be seen in the box to the left of the figure and in Fig 5). Plots in the same column have the same underlying digraph (from left to right, Directed Ring, Scale-Free, Small-World, Complete). All the simulations were performed for 50 time steps and 100 agents.

Fig 8 shows 12 different **IOTE** plots, for three different choices of the agent parameters (corresponding to the first, second and fourth histograms in Fig 5) and four different underlying digraphs. These plots reveal that the observations stemming from Fig 6 hold for opinion evolutions starting from any initial opinion distribution. It is also interesting to note that the parametric curves for the plot in the third row and second column (Scale-Free digraph with highly susceptible agents) do not immediately move towards the lines $\bar{x}=\pm \bar{\left|x\right|}$ (associated with perfect consensus), differently from what happens in the other plots with the same agent parameters (same row). This may be explained by the structure of the Scale-Free graph: in contrast to the other digraphs, which are composed of a single ‘highly connected entity’, the Scale-Free graph is composed of the interconnection of several ‘highly connected compartments’, and this peculiar topology slows down the tendency of the Friedkin-Johnsen model, with high agent susceptibility, to create perfect consensus.

**Fig 9:**
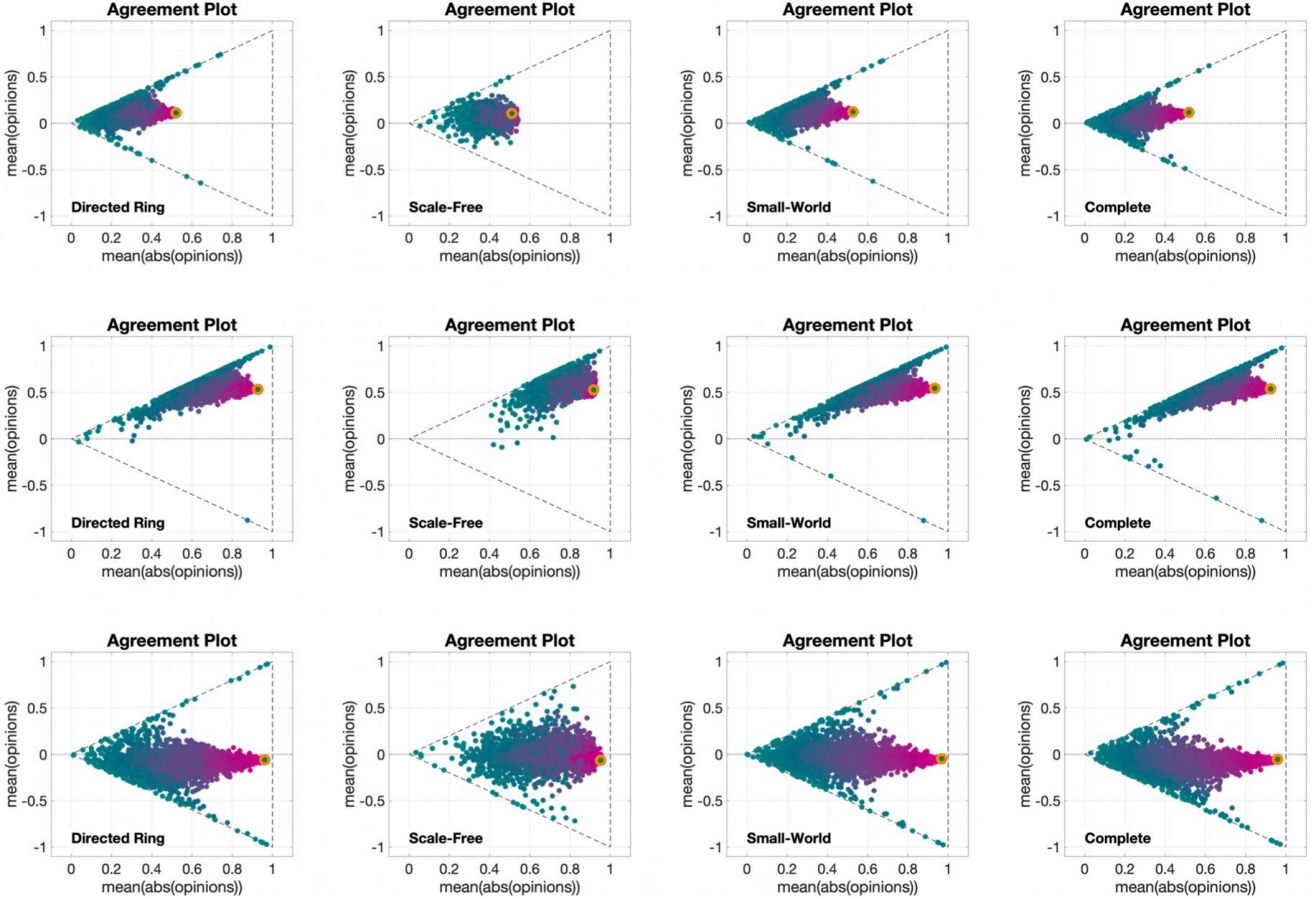
APSS plots for the Friedkin-Johnsen model. Each of the 12 plots includes 3528 points associated with different choices of the agent parameters, all with the same initial opinion distribution and underlying digraph. Plots in the same row have the same initial opinion distribution (orange circle). Plots in the same column have the same underlying digraph (from left to right, Directed Ring, Scale-Free, Small-World, Complete). All the simulations were performed for 1000 time steps and 100 agents.

The 12 different **APSS** plots in Fig 9, for three different choices of the initial opinion distribution (orange circles) and four different underlying digraphs, show that the qualitative effect of increasing the average susceptibility is the same for all initial opinions and digraphs. When the average susceptibility is low (magenta points), the final opinion distribution remains close to the initial one, as expected. As the average susceptibility increases, the points form a ‘cone’ to the left of the initial point. The higher the average susceptibility, the more the final opinion distributions move to the left. This trend continues until they arrive to the $\bar{x}=\pm \bar{\left|x\right|}$ lines and then move along these lines, which is where most of the teal (high susceptibility) points can be found. Hence, the higher the average susceptibility, the more likely the final opinions are to form perfect consensus, which is not surprising. What is surprising, is the noticeable difference between the second column and the others (as in Fig 6): with the Scale-Free digraph, the opinions do not change as much as with the other topologies. This is particularly evident in the plots of the third row, where even the teal points mostly do not reach the $\bar{x}=\pm \bar{\left|x\right|}$ lines.

**Fig 10:**
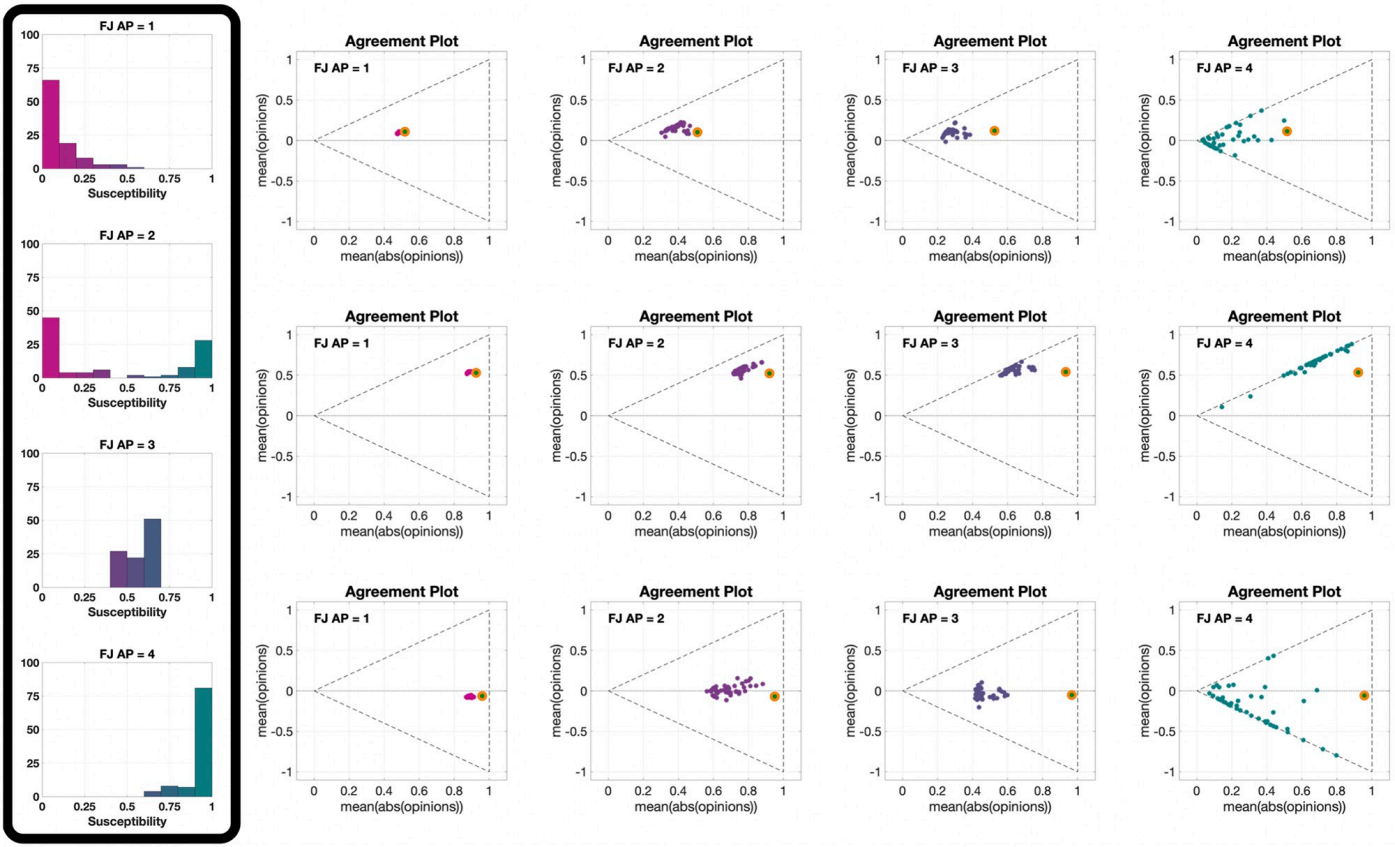
UDSS plots for the Friedkin-Johnsen model. Each of the 12 plots includes 45 points with different choices of underlying digraphs, all with the same initial opinion distributions and agent parameters. Plots in the same row have the same initial opinion distribution (orange circle). Plots in the same column have the same agent parameters (the histograms of these agent parameters can be seen in the box to the left of the figure and in Fig 5). All the simulations were performed for 1000 time steps and 100 agents.

The 12 **UDSS** plots in Fig 10, for three different initial opinions and four different agent parameters, confirm the observation from Fig 7 that societies where most agents have approximately the same susceptibility change more than societies with a similar mean susceptibility but with higher susceptibility variance. This can be seen by comparing the plots in columns 2 and 3 of Fig 10. It is also important to recall that the simulations seen in Fig 10 are for 1000 time steps, indicating that points closer to the initial opinion not only change slower, but also change less in the asymptotic behaviour.

**Fig 11:**
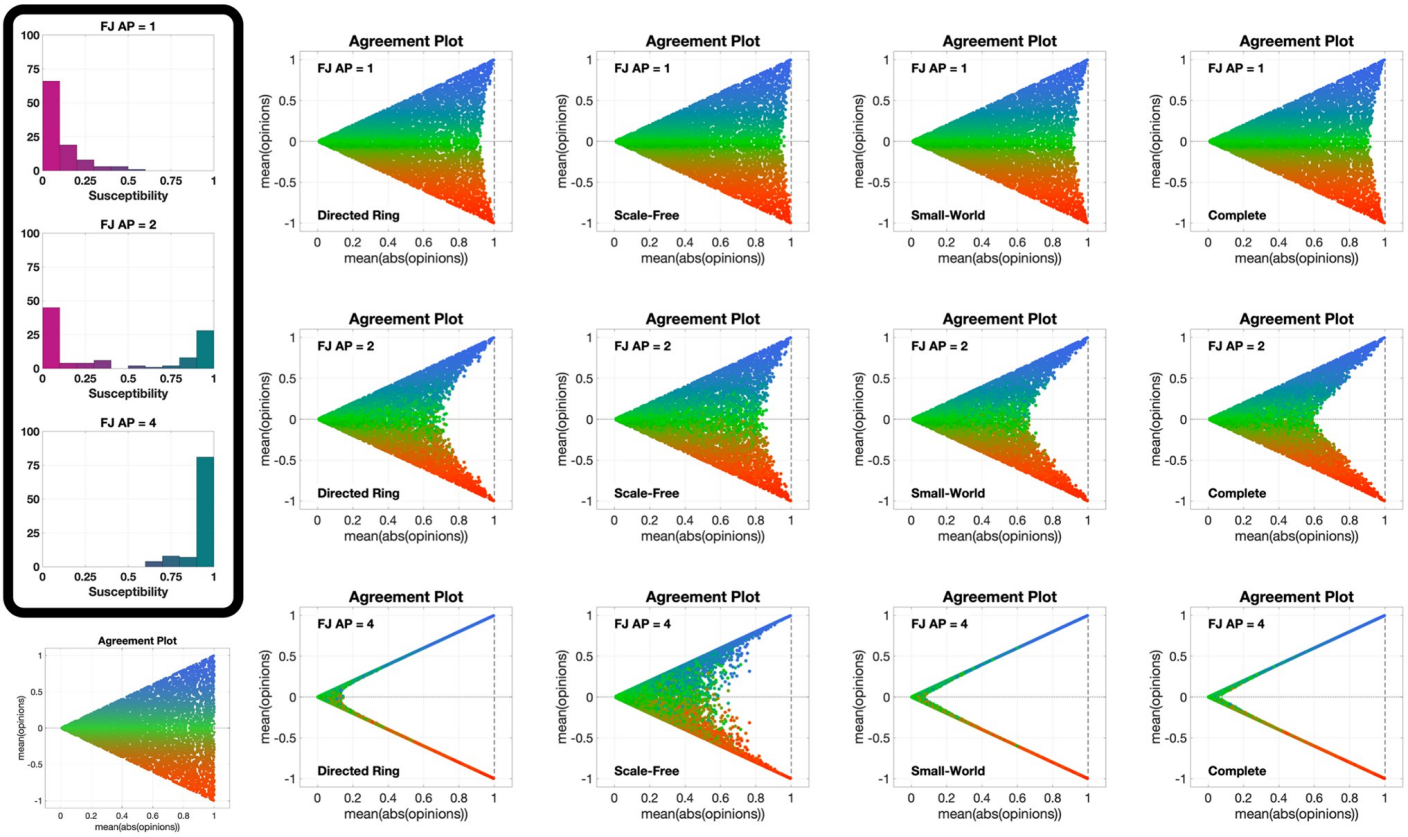
IOSS plots for the Friedkin-Johnsen model. Each of the 12 plots includes 5314 points associated with different choices of the initial opinion distributions, all with the same agent parameters and underlying digraph. Plots in the same row have the same agent parameters (the histograms of these agent parameters can be seen in the box to the left of the figure and in Fig 5). Plots in the same column have the same underlying digraph (from left to right, Directed Ring, Scale-Free, Small-World, and Complete). The reference plot for the location of the initial opinions, also shown in Fig 4C, can be seen in the bottom-left corner. All the simulations were performed for 1000 time steps and 100 agents.

Finally, Fig 11 presents 12 different **IOSS** plots showing how the reference initial opinion distributions in Fig 4B evolve for three different choices of the agent parameters (corresponding to the first, second and fourth histograms in Fig 5) and four different underlying digraphs. All the plots exhibit the contraction phenomenon initially seen in Fig 4D, and comparing the plots reveals an interesting nonlinearity in the ‘contraction factor’. In fact, even though the agent parameters in the second row represent the ‘middle point’ between the agent parameters in the first and third rows, the difference between the plots in the first and second rows is much smaller than the difference between the plots in the second and third rows. Regarding the underlying digraph, the Scale-Free digraph again yields a significantly different behaviour: it significantly reduces the contraction (by slowing down the convergence). The effect is barely visible in the first row (the outcome is only slightly affected by the digraph topology when the agents have very low average susceptibility), while it is more pronounced in the second row, and extremely pronounced in the third.

Overall, our graphical analysis allows us to draw the following conclusions on the opinion formation behaviour induced by the Friedkin-Johnsen model:

- Across different initial opinions, agent parameters, and underlying digraph, the opinions show the initial tendency to move towards the lines $\bar{x}=\pm \bar{\left|x\right|}$, corresponding to perfect consensus.
- When the average susceptibility is below about 0.5, or when the underlying digraph has the Scale-Free topology, the opinions change more slowly and the final opinion distribution is closer to the initial one.
- As the average susceptibility increases, or the average path length and diameter of the underlying digraph decrease, the opinions change faster and the final opinion distribution is closer to $\bar{x}=\pm \bar{\left|x\right|}$.
- When the average susceptibility is high, or the average path length and diameter of the underlying digraph are low, the opinions reach $\bar{x}=\pm \bar{\left|x\right|}$ and then move along either of these lines.
- The final opinion distribution is very unlikely to have a higher average of the opinion absolute values than the initial one; when that happens, either all the agents agree or they all disagree.
- The digraph topology appears to have a limited effect on the overall behaviour of the social systems.

### Analysis of the Classification-based model

**Fig 12:**
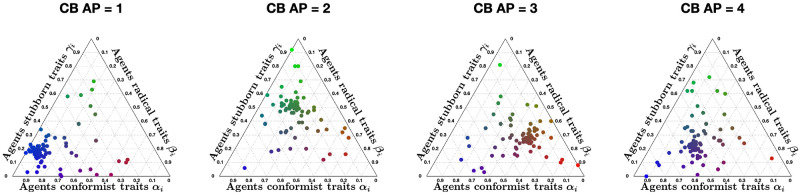
Ternary diagrams of different agent parameter choices for the Classification-based model with *N* = 100 agents. The diagrams were produced using modified scripts from Carl Sandrock [64]; details on how to interpret the ternary diagrams can be found in the S1 File. For the Classification-based model, each agent is characterised by three weights between 0 and 1, associated with conformism, radicalism and stubbornness, which add up to 1. We associate with each trait a colour: blue for conformism, red for radicalism, green for stubbornness. Then, the point corresponding to agent *i* has the RGB colour corresponding by *α*_*i*_ red, *β*_*i*_ blue and *γ*_*i*_ green. For instance, teal indicates an equal amount of conformism and stubbornness and very low radicalism, while red indicates a high level of radicalism and blue a high level of conformism.

We now adopt the graphical analysis to the Classification-based model [63], for several different choices of the agent parameters (conformism, radicalism and stubbornness, see Fig 12 for some examples), four different underlying digraph topologies (whose metrics are reported in Table 2) and several different initial conditions.

**Fig 13:**
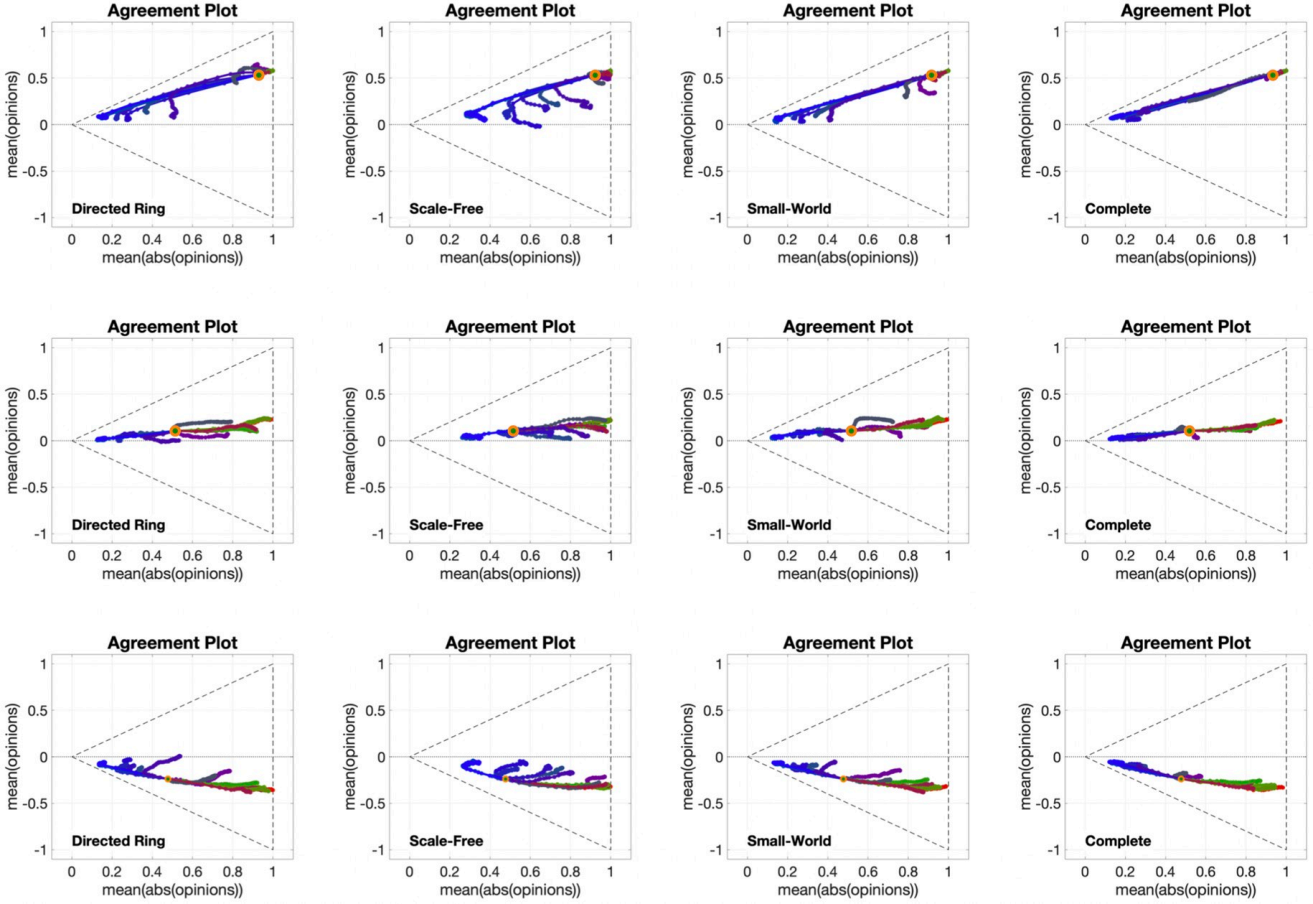
APTE plots for the Classification-based model. Each of the 12 plots includes 15 curves associated with different choices of the agent parameters, all with the same initial opinion distribution and underlying digraph. Plots in the same row have the same initial opinion distribution (orange circle). Plots in the same column have the same underlying digraph (from left to right, Directed Ring, Scale-Free, Small-World, Complete). All the simulations were performed for 50 time steps and 100 agents.

Fig 13 shows 12 different **APTE** plots, for three different choices of the initial opinion distribution (orange circles) and four different underlying digraphs. In these plots, we observe that the parametric curves can not only move to the left, but also to the right; also, for some initial opinions, some of the curves move towards the centre of the Agreement Plot. The colour coding reveals that the curves moving to the left (the standard behaviour with the Friedkin-Johnsen model) are mainly blue, representing highly conformist populations: in fact, in the Classification-based model the conformist trait produces consensus (as does susceptibility in the Friedkin-Johnsen model). On the other hand, the curves moving to the right are mostly red and green (but all with a red component): radicalism pushes the agent opinions to the extremes, resulting in a higher average of the absolute values of the opinions, and the effect can be seen even in the presence of a mild radical trait. Regarding noticeable differences due to the underlying digraph, curves associated with the Scale-Free digraph have a higher tendency to move towards the centre of the Agreement Plot, while in the other cases (and in particular for the Complete digraph) the curves move more to the right or to left. A possible explanation is that, since the Classification-based model allows for differences between the expressed opinion and how it is perceived by influenced neighbours, in graphs with longer average path length these discrepancies accumulate, thus diluting the trait’s effects and for instance preventing a highly conformist population from reaching consensus (so that even blue curves are not reaching the $\bar{x}=\pm \bar{\left|x\right|}$ lines).

**Fig 14:**
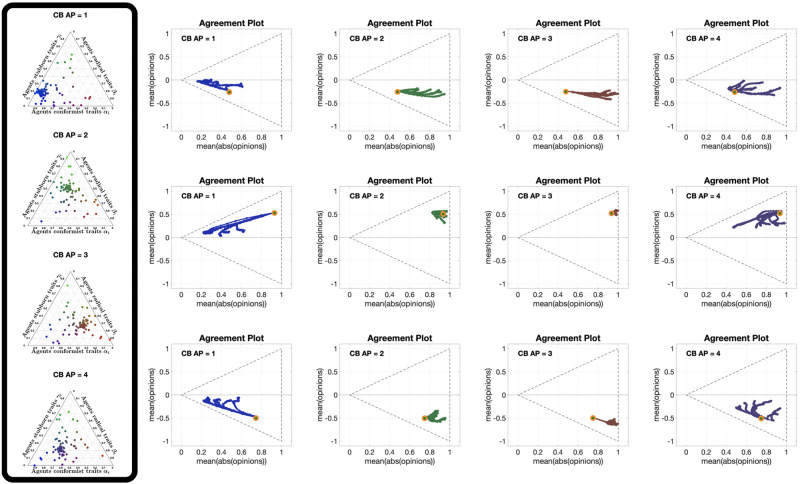
UDTE plots for the Classification-based model. Each of the 12 plots includes 45 curves with different choices of underlying digraphs (see Fig 3), all with the same initial opinion distributions and agent parameters. Plots in the same row have the same initial opinion distribution (orange circle). Plots in the same column have the same agent parameters (the ternary diagrams of these agent parameters can be seen in the box to the left of the figure and in Fig 12). All the simulations were performed for 50 time steps and 100 agents.

Fig 14 shows 12 **UDTE** plots, for three different initial opinions and four different choices of the agent parameters. These plots suggest that the agent parameters have a strong effect on the overall opinion evolution: regardless of the underlying digraph topology, the curves in each plot appear to be moving towards the same regions in the Agreement Plot. The directions of these movements are consistent with the observations made for the **APTE** plots: blue curves move generally towards the left of the Agreement Plot, red and green curves move to the right, and a more equal combination of the three colours moves to the centre-right. Taking into account that all the curves in a given plot move in the same direction, it is possible to conclude that the underlying digraph has an indirect influence on the opinion evolutions that cannot prevail over the effect of the agent parameters.

**Fig 15:**
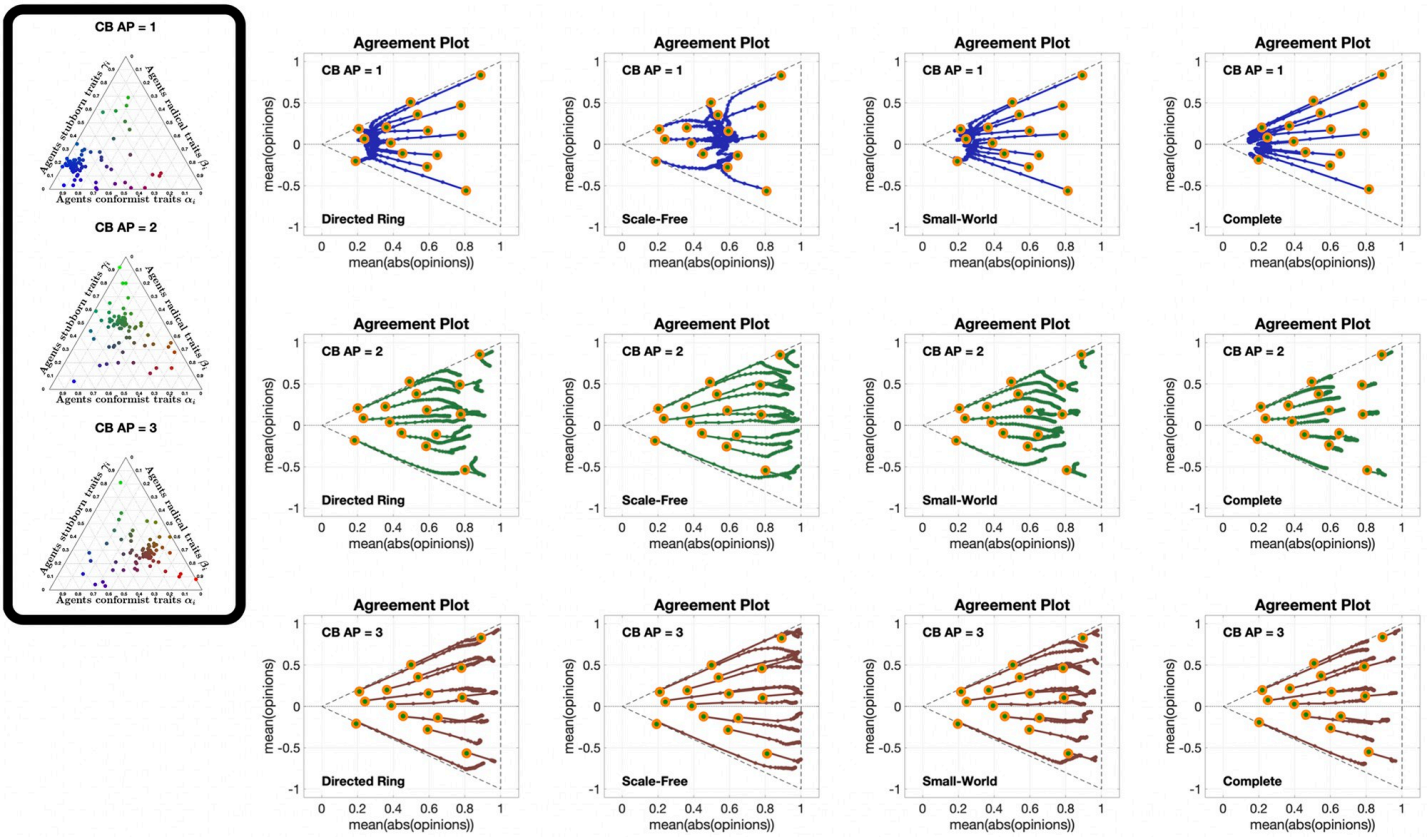
IOTE plots for the Classification-based model. Each of the 12 plots includes 15 curves associated with different choices of the initial opinion distributions, all with the same agent parameters and underlying digraph. Plots in the same row have the same agent parameters (the ternary diagrams of these agent parameters can be seen in the box to the left of the figure and in Fig 12). Plots in the same column have the same underlying digraph (from left to right, Directed Ring, Scale-Free, Small-World, Complete). All the simulations were performed for 50 time steps and 100 agents.

Fig 15 shows 12 different **IOTE** plots, for three different choices of the agent parameters (corresponding to the first three ternary diagrams in Fig 12) and four different underlying digraphs. These plots reveal a new behaviour: given a choice of the agent parameters and of the underlying digraph, all the opinion distributions move towards the same region in the Agreement Plot, regardless of the initial condition. While for the Friedkin-Johnsen model all the curves moved in the same direction, for the Classification-based model the direction followed by each curve depends on the initial opinion location in the Agreement Plot and on the region to which all curves converge (which is just determined by the agent parameters and the underlying digraph). The convergence speed (inferred from the distance between consecutive points in the same parametric curve) seems to depend only on the agent parameters and not on the underlying digraph: the distance is smaller for the green curves, indicating that populations with mostly stubborn traits change their opinion more slowly. This is consistent with the stubborn trait’s tendency to keep the opinions unchanged, and thus slow down the opinion change caused by the other traits.

**Fig 16:**
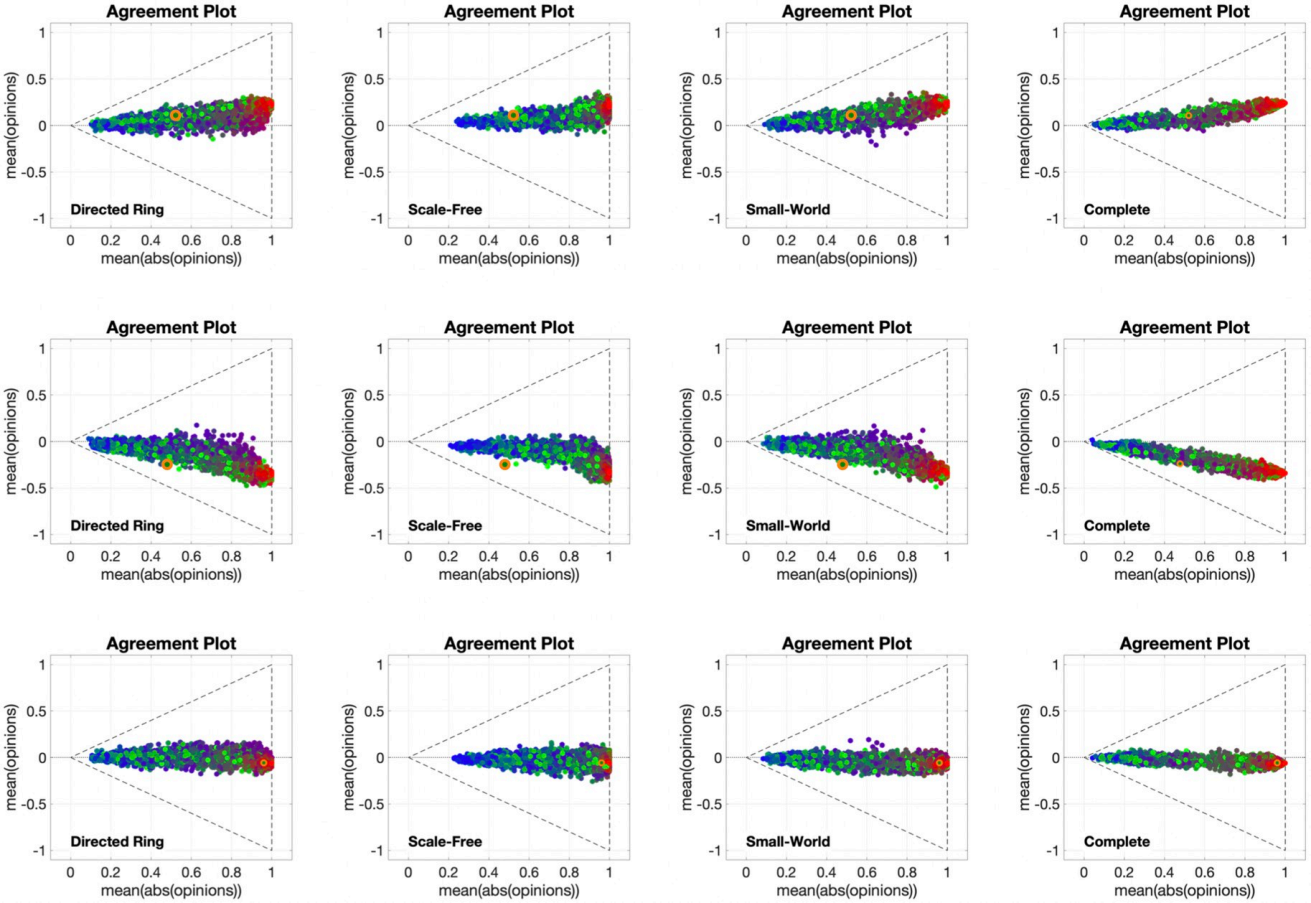
APSS plots for the Classification-based model. Each of the 12 plots includes 3528 points associated with different choices of the agent parameters, all with the same initial opinion distribution and underlying digraph. Plots in the same row have the same initial opinion distribution (orange circle). Plots in the same column have the same underlying digraph (from left to right, Directed Ring, Scale-Free, Small-World, Complete). All the simulations were performed for 1000 time steps and 100 agents.

The 12 different **APSS** plots shown in Fig 16, for three different choices of the initial opinion distributions (orange circles) and four different underlying digraphs, look remarkably different from the same type of plots for the Friedkin-Johnsen model. In fact, few points are located along the lines $\bar{x}=\pm \bar{\left|x\right|}$, showing that consensus is not the most likely outcome. The points tend to form a connected (and in most cases convex) subset of the Agreement Plot. The initial opinion distribution is sometimes in the interior of the region, sometimes at the boundary. Unlike the Friedkin-Johnsen model, the Classification-based model seems to yield a narrow range of opinion distributions, once the underlying graph and the initial condition have been fixed. In particular, most final opinion distributions have an ordinate that is not too far from that of the initial opinion distribution: the Classification-based model evolution does not strongly affect the average of the opinions. Also, looking at the colour coding, the points to the right are mostly red, those to the left are mostly blue, while green points are close to the initial opinion distribution point in either direction: not surprisingly, predominantly radical societies tend to more extreme opinions, predominantly conformist societies tend to less extreme opinions, while predominantly stubborn societies do not change much and the change direction is determined by the trait with the second highest weight.

**Fig 17:**
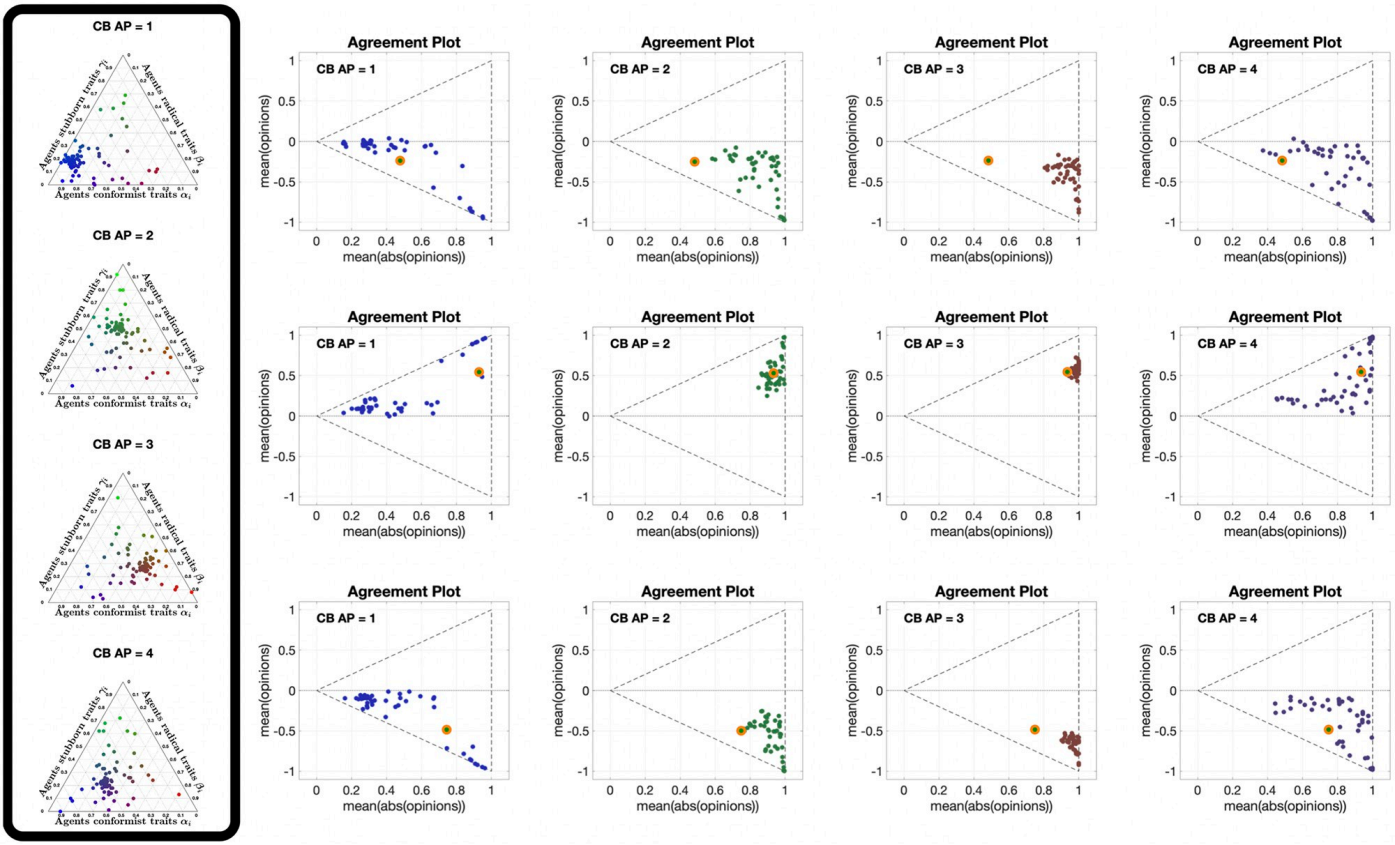
UDSS plots for the Classification-based model. Each of the 12 plots includes 45 curves with different choices of underlying digraphs, all with the same initial opinion distributions and agent parameters. Plots in the same row have the same initial opinion distribution (orange circle). Plots in the same column have the same agent parameters (the ternary diagrams of these agent parameters can be seen in the box to the left of the figure and in Fig 12). All the simulations were performed for 1000 time steps and 100 agents.

Fig 17 shows 12 **UDSS** plots for three different initial opinions and four different choices of the agent parameters. Like the **APSS** plots, the **UDSS** plots for the Classification-based model are very different from the ones obtained for the FJ model. The plots in Fig 17 indicate that, for the Classification-based model, the digraph topology has a more direct and clear effect on the agents’ opinion evolution. For instance, looking at column 1, we can see that, even for a highly conformist society, for some underlying topologies extreme opinions can appear (represented by dots near the *x* = 1 line), thus conformism alone is not enough to prevent extreme opinions from forming. Dots in columns 2 and 4 are also very scattered and in many cases can be found around the initial opinion point, further implying that even for a fixed agent parameter set, the digraph topology has a significant effect on the opinion evolution.

The only parameters for which this effect is somewhat less evident are the ones in column 3, where most of the dots are located to the right of the initial opinions distribution dot. This may suggest that the radical trait in the Classification-based model has a more powerful effect than the other traits, overshadowing the influence of the underlying digraph topology.

**Fig 18:**
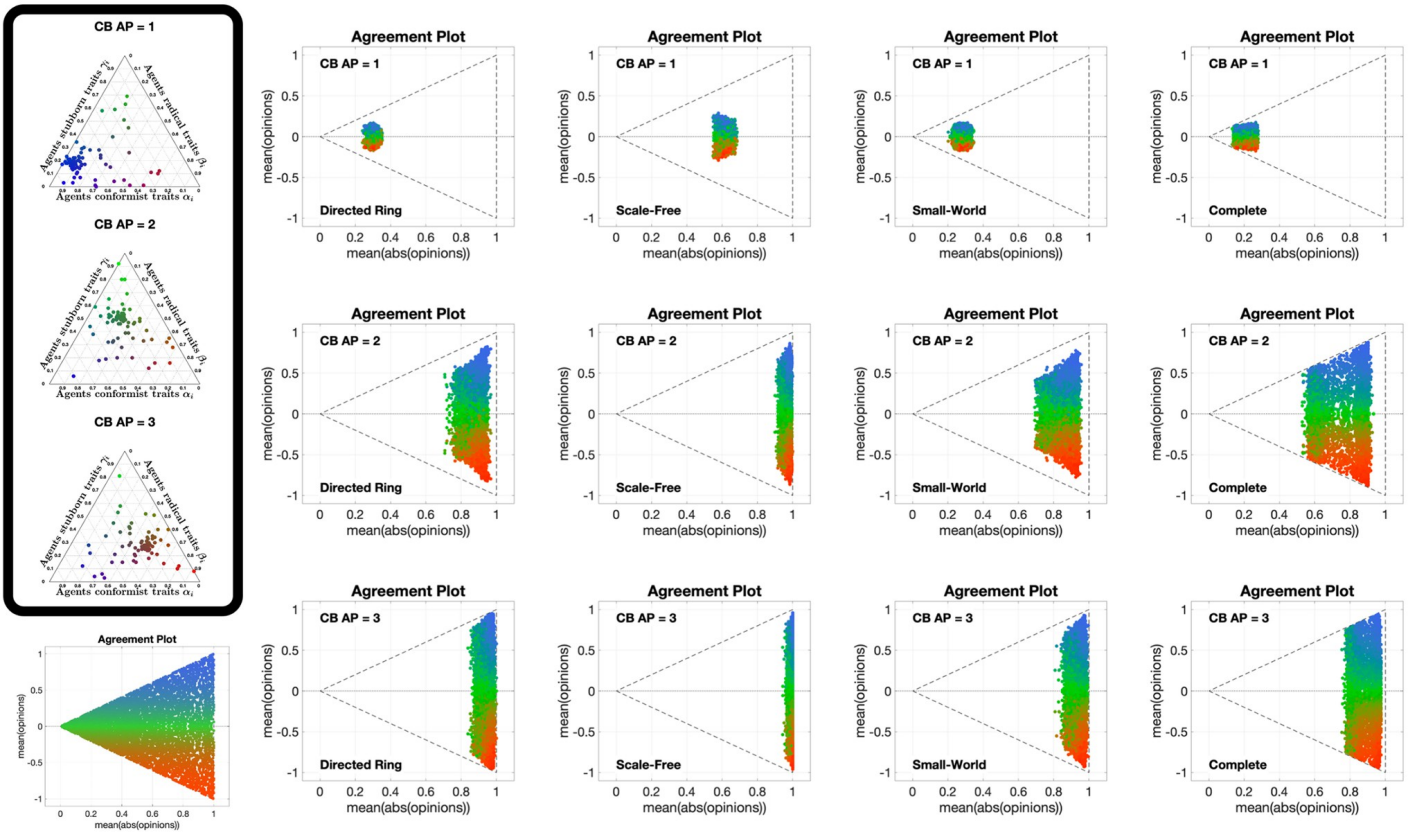
IOSS plots for the Classification-based model. Each of the 12 plots includes 5314 points associated with different choices of the initial opinion distributions, all with the same agent parameters and underlying digraph. Plots in the same row have the same agent parameters (the ternary diagrams of these agent parameters can be seen in the box to the left of the figure and in Fig 12). Plots in the same column have the same underlying digraph (from left to right, Directed Ring, Scale-Free, Small-World, and Complete). The reference plot for the location of the initial opinions, also shown in Fig 4C, can be seen in the bottom-left corner. All the simulations were performed for 1000 time steps and 100 agents.

The 12 different **IOSS** plots in Fig 18, for three different choices of the agent parameters (corresponding to the first three ternary diagrams in Fig 12) and four different underlying digraphs, confirm the existence of ‘convergence regions’ towards which all the opinions move. As in Fig 15, the shape and location of these regions depend on the agent parameters and underlying digraph. Also, the regions are symmetric with respect to the axis $\bar{x}=0$ (the model does not encode any preference for agreement vs. disagreement) and are mostly located away from the boundary of the triangle, consistently with Fig 16. Moreover, there are no azure points with a negative $\bar{x}$ and there are no orange points with a positive $\bar{x}$: the average of the opinions does not change much, as previously noticed.

Our graphical analysis has highlighted the behaviour and intrinsic properties of the Classification-based model as follows:

- Regardless of the initial conditions, the curves in the Agreement Plot move towards a ‘convergence region’ whose location and shape depends on the agent parameters and the underlying digraph. The convergence region is often detached from the boundaries of the (0, 0), (1, −1), (1, 1) triangle.
- Although the agent parameters have a significant effect in the opinion evolution, the final opinion distribution highly depends on the underlying digraph, much more than with the Friedkin-Johnsen model.
- For the considered underlying digraphs and initial opinions, the collection of possible opinion outcomes obtained with different agent parameters forms a connected (and mostly convex) region in the Agreement Plot that contains the initial opinion distribution.
- Final opinion distributions are rarely located near the lines $\bar{x}=\pm \bar{\left|x\right|}$ and $\bar{\left|x\right|}=1$: almost always, some agents agree while others disagree, and outcomes where all the agents have extreme opinions are quite uncommon.

## Conclusions

We have proposed a novel methodology to analyse deterministic agent-based opinion formation models. Our technique relies on the **Agreement Plot**, which captures several opinion distributions in the same Cartesian plane, thus allowing for a thorough and systematic visual comparison of the opinion evolution achieved with different initial opinions, agent parameters, and underlying digraphs. This comparison reveals if and how global patterns produced by the considered model relate to the specific model features, and can also unveil intrinsic properties of the model.

We have used the **Agreement Plot** as a canvas to display either parametric curves of the opinion evolution over time or points associated with the final opinion distribution, for different agent parameters and different initial opinion distributions, resulting in six types of plots: **APTE**, **UDTE** and **IOTE**, focused on the time evolution for different agent parameters, different underlying digraphs and different initial opinion distributions respectively, as well as **APSS**, **UDSS** and **IOSS**, focused on the steady-state opinion distribution for different agent parameters, different underlying digraphs and different initial opinion distributions respectively. By systematically varying the choice of the agent parameters, the underlying digraph topology, and the initial opinion distribution, we could gain a deeper insight into how the opinion formation process relates to these properties of the model, and understand which patterns are intrinsic to the model and which depend on the chosen model features.

We conducted our graphical analysis for the Friedkin-Johnsen model [29, 30] and the recently proposed Classification-based model [63]. For the Friedkin-Johnsen model, the analysis reveals that: (i) when the average path length and diameter of the underlying digraph decrease, the opinions change faster, and the final opinion distribution is closer to perfect consensus; (ii) when the average susceptibility is high, and the average path length and digraph diameter are low, the opinions tend to reach perfect consensus; (iii) the final opinion distribution rarely has a higher average of the opinion absolute values than the initial one, and, if this happens, the opinions form perfect consensus. For the Classification-based model, conversely, the analysis reveals that: (i) opinion distributions move to a ‘convergence region’ that depends on the agent parameters and the underlying digraph, regardless of the initial conditions; (ii) the final predicted opinions are highly dependent on the underlying digraph, much more than for the Friedkin-Johnsen model; (iii) outcomes where all agents have extreme opinions are rare; (iv) a wide variety of outcomes can be obtained by changing the initial conditions, the agent parameters and the underlying digraph topology.

The proposed methodology is not however, without some limitations. By representing the opinion of (possibly) thousands of agents as a single point in the Cartesian plane, relevant information is highlighted, but, unavoidably, some information is lost. In some cases, the missing information may lead to misinterpretation of the results and wrong conclusions.

For instance, the representation may be misleading in terms of information compression, because qualitatively distant macroscopic configurations are encoded as similar or identical points in the Agreement Plot. Consider for instance two populations, one where the opinions are uniformly distributed, and another one where half of the population has opinion 0.5 and the other half has opinion −0.5. Both these populations are represented by the same point in the Agreement Plot, while having qualitatively distinct macroscopic configurations. In the first case, every opinion is held by the same number of agents and no one opinion is prevalent. In the second case, only two opinions are possible and there is perfect polarisation in the society. These types of representation level misinterpretations are more common for points near the centre of the Agreement Plot.

Misinterpretation may also occur at the level of model analysis. For example, consider the case of a network of two agents with a mutual interconnection that continually swap their initial opposite opinions of 1 and −1, because they evolve according to Friedkin-Johnsen dynamics and have complete susceptibility and no self-confidence. In this example, both agents in the population undertake radical and continuous opinion changes; however, the corresponding parametric curve would be a single point in the Agreement Plot, potentially leading to the misleading interpretation that the system may have reached an equilibrium. As we have emphasised, though, the fact that the representation in the Agreement Plot remains the same does not mean that the actual opinion distribution is also unchanged.

Although the loss of distributional information about the opinions is unavoidable when using this technique, some alternatives may be explored to offer new interpretations or decrease the risk of misinterpretation, by incorporating information about the network topology in the calculation of *π*(*x*) in Eq (1) (e.g., by accounting for opinion agreement only between agents that are connected by an edge in the digraph). For instance, a weight reflecting the agent’s degree in the network may reflect the effect of the opinion of an agent in the overall population. It would be interesting to compare Agreement Plots produced based on different modified definitions of *π*(*x*) and try to find invariants in different models.

Our proposed technique can be applied to analyse the behaviour of any agent-based opinion formation model in which the agent opinions belong to a bounded interval in the real line, and provides a non-conventional perspective to study opinion formation models. In a graphical and intuitive way, it showcases how the opinion evolution depends on the model parameters and also what the model capabilities are, thanks to the representation of a complete opinion distribution as a single point in the Cartesian plane (which of course leads to the loss of some information, but keeps and highlights the most relevant information about the collective behaviour).

When applying the technique, a critical parameter to consider is the minimum number of time steps of the simulation horizon that are needed for the system to reach a steady state. This value can be determined, during a preliminary analysis of the general properties of the considered model, either theoretically or via comprehensive numerical simulations. For a system that converges asymptotically, it is sufficient to only simulate to an *ϵ*-neighbourhood of the steady state. Even in the absence of convergence, our technique could still be applied for different simulation horizons (plotting only the final simulated state) and comparing the results. These could be thought of as ‘snapshots’ of a constantly changing system. The usefulness of this technique is not bound to simulating until a steady state is achieved: it can also reveal global patterns of agent-based models that are allowed to evolve for a given period of time.

The proposed methodology is flexible enough to be modified in order to conduct other analyses of interest (e.g. adding a third dimension or modifying the colour coding to represent an additional relevant metric), and can be used not only to study the properties of individual opinion models, but also to compare existing opinion formation models, which has been acknowledged as one critical aspect in the study of opinion formation models [8].

As a final observation, the Agreement Plots, besides offering visual semi-quantitative insight as discussed in this paper, can help inform and design specific statistical tests for subsequent investigations that can provide quantitative conclusions on the differences between agent-based models (for instance, investigating the effects of different network topologies, or of external influences on the population).

## Code availability

All our code is available at https://giuliagiordano.dii.unitn.it/docs/papers/GAcode.zip.

## Supporting information

S1 File The SI file provides: A brief explanation on how to use our code to reproduce the results presented in the paper; a description of the considered agent-based opinion formation models; details on the computation of the considered graph metrics; the application of the proposed graphical analysis methodology to two additional models, the Bounded Confidence model and the Backfire Effect and Biased Assimilation model; and higher resolution images for the result figures. (PDF)
