# Supplementary material for: Graphical analysis of agent-based opinion formation models
Source: PLoS One. 2024 May 30;19(5):e0303204. doi: 10.1371/journal.pone.0303204 (PMC11139329; doi:10.1371/journal.pone.0303204)
Supplement: S1 File — (PDF) [file pone.0303204.s001.pdf]

Graphical Analysis of Agent-Based Opinion Formation Models
Supplementary Information

Carlos Andrés Devia[1] and Giulia Giordano[1,2,*]

[1]Delft Center for Systems and Control, Delft University of Technology, 2628 CD Delft, The Netherlands
[2]Department of Industrial Engineering, University of Trento, 38123 Trento, Italy
[*]g.giordano@tudelft.nl

Abstract

This Supplementary Information for the paper *Graphical Analysis of Agent-Based Opinion Formation Models* provides: a brief explanation on how to use our code to reproduce the figures presented in the Main Paper; a description of the considered agent-based opinion formation models; details on the computation of the considered graph metrics; the application of the proposed graphical analysis methodology to two additional models, the Bounded Confidence model and the Backfire Effect and Biased Assimilation model; and higher resolution images for the result figures in the Main Paper.

Contents

1 Code usage **1**

2 Agent-based opinion formation models **1**
2.1 Classification-based opinion formation model 2
2.2 Bounded Confidence model 3
2.3 Friedkin-Johnsen model 3
2.4 Backfire Effect and Biased Assimilation model 3

3 Graph metrics **4**

4 Agreement Plot analysis of additional models **5**
4.1 Backfire Effect and Biased Assimilation model 5
4.2 Bounded Confidence model 13

References **19**

A High-resolution images for figures in the Main Paper **20**
A.1 Friedkin-Johnsen model 20
A.2 Classification-based model 25

1 Code usage

The Matlab code used to conduct the analysis and produce the figures presented in the paper is available at:
`https://giuliagiordano.dii.unitn.it/docs/papers/GAcode.zip`

All the code is executed by running the script `GlobalScript_AgreementPlot.m`. All the necessary files and scripts are included in the .zip file and executing the code requires about 5 hours. The .zip file also contains a README file, with more detailed instructions on how to use the code.

The ternary diagrams were produced using modified scripts from Carl Sandrock [1].

2 Agent-based opinion formation models

We provide a brief description of the models analysed in the Main Paper and in the Supplementary Information.

2.1 Classification-based opinion formation model

In the Classification-based (CB) model [2], the *opinion* of agent i at time k represents its level of agreement with a statement and is denoted by $x_i[k] \in [-1, 1]$, where $i \in \mathcal{V}$ and the set $\mathcal{V} = \{1, 2, \ldots, N\}$ indexes the agents. The opinions $x_i = 1$, $x_i = 0$, and $x_i = -1$ represent complete agreement, indifference, and complete disagreement respectively. The vector of all the agent opinions at time k is denoted by $x[k]$.

The agent opinions evolve in discrete time over a signed digraph, represented by the matrix $W \in \{-1, 0, 1\}^{N \times N}$. The weight w_{ij} represents the influence of agent j over agent i. If $w_{ij} = 0$, then agent i is not influenced by agent j. If $w_{ij} \neq 0$, then agent j is a neighbour of agent i: $w_{ij} = 1$ means that agent i approves, trusts, or follows agent j, while $w_{ij} = -1$ means that agent i disapproves, mistrusts, or antagonises agent j. The self-confidence of each agent is expressed by $w_{ii} = 1$ for all i. In the model, if $w_{ij} = 1$ (respectively $w_{ij} = -1$), then agent i perceives the opinion of agent j as x_j (resp. $-x_j$). The set of neighbours of agent $i \in \mathcal{V}$ is

$$\mathcal{N}_i = \Big\{ j \in \mathcal{V} \colon w_{ij} \neq 0 \Big\}. \tag{1}$$

The model evolves according to the equation

$$x_i[k+1] = \sigma\left(x_i[k] + \frac{\lambda}{|\mathcal{N}_i|} \Big(\alpha_i \xi \big(|A_i^+| - |D_i^+|\big) + \alpha_i \big(|A_i| - |D_i|\big) + \beta_i \mu |N_i| x_i[k] \Big) \right), \tag{2}$$

for all $i \in \mathcal{V}$, where

- $\sigma(x) = \max\{-1, \min\{1, x\}\}$ is a saturating function;
- λ, ξ, and μ are model parameters with values 0.4, 2, and 5, respectively (see [2] for more details);
- the *agent parameters* α_i, β_i, γ_i (*inner traits of agent* i, collectively denoted as ψ_i) belong to the set

  $$(\alpha_i, \beta_i, \gamma_i) \in \Sigma = \Big\{ \alpha_i, \beta_i, \gamma_i \in [0, 1] \quad \text{and} \quad \alpha_i + \beta_i + \gamma_i = 1 \Big\}$$

  and represent the weight of the three inner traits that characterise every agent: conformism (α_i), radicalism (β_i), and stubbornness (γ_i); the conformist trait tends to decrease the opinion distance between an agent and its neighbours, the radical trait moves the opinion of an agent to the extreme, and the stubbornness trait prevents opinion change;
- the subsets D_i^+, D_i, N_i, A_i, and A_i^+ yield a time-varying partition of the set $\mathcal{N}_i$ of all the neighbours of agent $i \in \mathcal{V}$, are defined as

  $$\begin{aligned} D_i^+[k] &= \{ j \in \mathcal{N}_i \colon 6/5 \leq \Delta_{ij}[k] \leq 2 \} \\ D_i[k] &= \{ j \in \mathcal{N}_i \colon 2/5 \leq \Delta_{ij}[k] < 6/5 \} \\ N_i[k] &= \{ j \in \mathcal{N}_i \colon -2/5 < \Delta_{ij}[k] < 2/5 \} \\ A_i[k] &= \{ j \in \mathcal{N}_i \colon -6/5 < \Delta_{ij}[k] \leq -2/5 \} \\ A_i^+[k] &= \{ j \in \mathcal{N}_i \colon -2 \leq \Delta_{ij}[k] \leq -6/5 \} \end{aligned} \tag{3}$$

  and represent the subsets of neighbours that *agree much less*, *agree less*, *agree comparably*, *agree more*, *agree much more* than agent i with the considered statement.

Since the values for λ, ξ, and μ are fixed, the model has two type of parameters:

- the **inner traits assignation** $\psi \in \mathcal{A}$, where $\mathcal{A}$ is the set of all possible inner trait assignations:

  $$\mathcal{A} = \Big\{ \psi = (\psi_i)_{i \in \mathcal{V}} = \big((\alpha_i, \beta_i, \gamma_i)\big)_{i=1}^N \colon (\alpha_i, \beta_i, \gamma_i) \in \Sigma \Big\}; \tag{4}$$

- the **underlying signed digraph**, represented by a weight matrix $W \in \mathcal{W}$. In general the set of all possible underlying signed digraphs is $\mathcal{W} = \{-1, 0, 1\}^{N \times N}$, but we can focus for instance on small-world, or strongly connected, graphs.

Given an inner trait assignation $\psi = (\psi_i)_{i \in \mathcal{V}} = \big((\alpha_i, \beta_i, \gamma_i)\big)_{i=1}^N$, we can compute the *average inner traits* $(\overline{\alpha}, \overline{\beta}, \overline{\gamma})$, which are the average weights within the population:

$$\bar{\alpha} = \frac{1}{N} \sum_{i \in \mathcal{V}} \alpha_i \qquad \bar{\beta} = \frac{1}{N} \sum_{i \in \mathcal{V}} \beta_i \qquad \bar{\gamma} = \frac{1}{N} \sum_{i \in \mathcal{V}} \gamma_i, \tag{5}$$

Both the inner traits of a single agent ψ_i and the average inner traits $\overline{\psi}$ can be visualised as a dot in a ternary diagram, as shown in Fig 1.

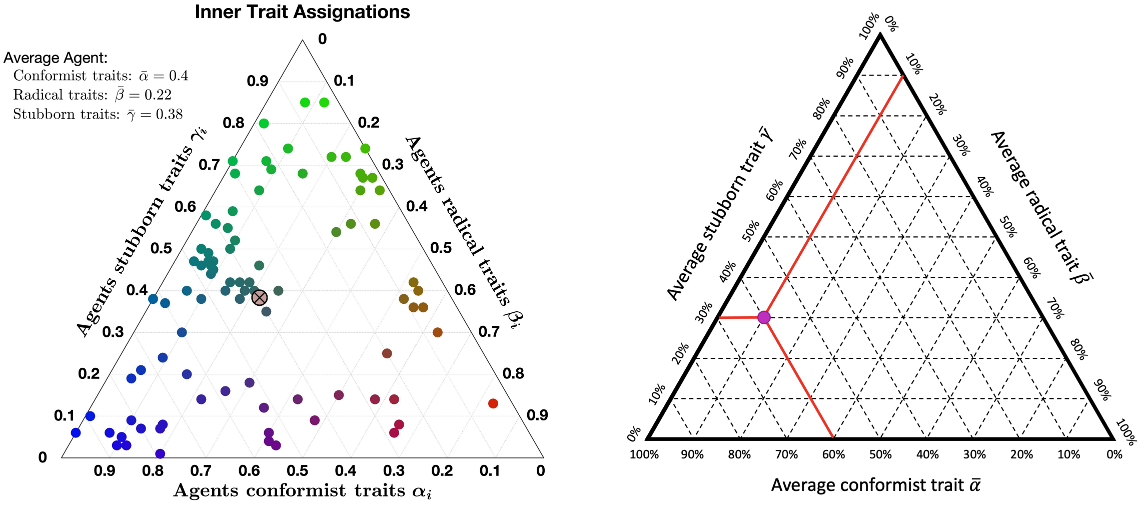

Figure 1: Ternary diagrams visualising inner traits assignations ψ and average inner traits $\bar{\psi}$. Left: the ternary diagram shows the whole inner traits assignation, where each dot represents the inner traits of a singe agent and its RGB colour reflects the weight of each trait (blue: conformist; red: radical; green: stubborn); the crossed dot represents the average inner traits. Right: the ternary diagram only shows the average inner traits of a complete population (magenta dot) that is 60% conformist, 10% radical, 30% stubborn.

2.2 Bounded Confidence model

The Bounded Confidence model [3] evolves according to the equation:

$$x_i[k+1] = \sum_{j \in \mathcal{V}} w_{ij}[k] x_j[k], \quad w_{ij}[k] = \begin{cases} \frac{1}{|N_i|} & \text{if } |x_i[k] - x_j[k]| \leq r_i \\ 0 & \text{otherwise} \end{cases} \qquad \forall i \in \mathcal{V},$$

where $N_i = \{j \in \mathcal{V} \colon |x_i[k] - x_j[k]| \leq r_i\}$, and $|N_i|$ is its cardinality. The Bounded Confidence model behaves as a French-DeGroot model [4, 5, 6, 7] where the digraph is time-varying: each agent is characterised by a *confidence radius* $r_i \in [0, 2]$ and can only be influenced by another agent when the difference between their opinions is smaller or equal to the confidence radius. At each time step, matrix W is still row-stochastic. Since edges can be created and removed throughout the dynamic evolution of the model, the whole graph topology changes with time, and not only the edge weights.

2.3 Friedkin-Johnsen model

The Friedkin-Johnsen model [8, 9] generalises the French-DeGroot model [4, 5, 6, 7] by including for each agent a parameter called *susceptibility*. The susceptibility of agent $i \in \mathcal{V}$ is denoted by $\lambda_i \in [0, 1]$. According to the Friedkin-Johnsen model, the opinion of agent i evolves as

$$x_i[k+1] = \lambda_i \Big(\sum_{j \in \mathcal{V}} w_{ij} x_j[k] \Big) + (1 - \lambda_i) u_i, \quad \forall i \in \mathcal{V},$$

where u_i is called the *prejudice* of agent i; we consider $u_i = x_i[0]$. If $\lambda_i = 1$ for all agents, then we get the French-DeGroot model. The digraphs over which the Friedkin-Johnsen model evolves are time-invariant and row-stochastic.

2.4 Backfire Effect and Biased Assimilation model

The Backfire Effect and Biased Assimilation model [10] also generalises the French-DeGroot model in order to include backfire effect and biased assimilation. Backfire effect occurs when, after agents i and j communicate, their resulting opinions are more distant than before their interaction. Biased assimilation is a form of homophily, in that it represents the tendency to be more influenced by neighbours with a similar opinion. Both these mechanisms are represented by the *entrenchment* parameter, a positive number associated with each agent: $\kappa_i > 0$. If κ_i is small, then the agent's backfire and biased assimilation tendencies are low, and as κ_i increases so does the strength of these tendencies. The opinion of agent i evolves as

$$x_i[k+1] = \begin{cases} \operatorname{sgn}(x_i[k]) & \text{if } \sum_{j \in \mathcal{V}} w_{ij}[k] \leq 0 \\ \frac{\sum_{j \in \mathcal{V}} w_{ij}[k] x_i[k]}{\sum_{j \in \mathcal{V}} w_{ij}[k]} & \text{otherwise} \end{cases}$$

and the edge weights evolve as

$$w_{ij}[k] = \begin{cases} \kappa_i x_i[k] x_j[k] + 1 & \text{if } w_{ij}[0] \neq 0, \\ 0 & \text{if } w_{ij}[0] = 0. \end{cases}$$

Hence, edges that were not present in the initial digraph cannot be added, and the edges that were initially present may change sign and weight (for some specific opinions, the edge could become zero, effectively removing the edge for that time step), meaning that the *topology* remains approximately the same.

3 Graph metrics

A signed digraph is represented by its weight matrix $W \in \{-1, 0, 1\}^{N \times N}$, where w_{ij} is associated with the edge going from vertex j to vertex i. Here, we discuss how to compute the following graph metrics: Average Path Length, APL; Diameter, D; Average clustering, $\overline{CC}$; Clustering variance, $\sigma(CC)$; Average out-degree, $\overline{\delta^{out}}$; Out-degree variance, $\sigma(\delta^{out})$; Average in-degree, $\overline{\delta^{in}}$; In-degree variance, $\sigma(\delta^{in})$; Number of edges, #E; Number of positive edges, #P; Number of negative edges, #N; Bidirectional Coefficient, Bc; Positive to negative edge ratio, +/-.

A directed path is a K-tuple of vertices $(p_1, p_2, \ldots, p_i, p_{i+1}, \ldots, p_K)$ such that there is an edge from vertex p_i to vertex p_{i+1} for $i = 1, \ldots, K-1$. The length $|p|$ of a directed path p is the number of edges that it crosses. Let $P(i,j)$ be the set of all directed paths from vertex i to vertex j (if there are none, then $P(i,j) = \emptyset$). Denote by $d(i,j)$ the length of the shortest directed path from i to j, i.e., $d(i,j) = \min_{p \in P(i,j)} |p|$. Let $C(W)$ be the set of vertex pairs (i,j) such that there exists a direct path from i to j and $i \neq j$, i.e. $C(W) = \{(i,j) \colon P(i,j) \neq \emptyset \text{ and } i \neq j\}$. Then the average path length and the diameter of the digraph W are:

$$APL = \frac{1}{|C(W)|} \sum_{(i,j) \in C(W)} d(i,j) \qquad \text{and} \qquad D = \max_{(i,j) \in C(W)} d(i,j). \tag{6}$$

Since all the graphs that we consider are strongly connected, $|C(W)| = N(N-1)$.

To compute the clustering coefficient, consider agent i, with k_i in-neighbours excluding itself: $k_i = |\tilde{\mathcal{N}_i}|$, where $\tilde{\mathcal{N}_i} = \{j \in \mathcal{V} \colon w_{ij} \neq 0, i \neq j\}$. Then there are at most $k_i(k_i - 1)$ directed edges between these neighbours. The fraction c_i of these edges that is actually present is the clustering coefficient of agent i. If agent i has only one in-neighbour, then its clustering coefficient is 1, and if it has no in-neighbour but itself c_i is not defined:

$$c_i = \begin{cases} \frac{|\{(j,k) \colon j \neq k \text{ and } i,k \in \tilde{\mathcal{N}_i}\}|}{k_i(k_i-1)} & \text{if } k_i > 1 \\ 1 & \text{if } k_i = 1 \\ \text{undefined} & \text{if } k_i = 0 \end{cases} \tag{7}$$

The average clustering and the clustering variance of the graph are thus, respectively, the average and the variance of the clustering coefficients of all the agents with at least one in-neighbour excluding themselves. The average clustering is also sometimes called clustering coefficient [11].

For the connectivity degree measures, consider a vertex i. The in-degree (respectively, out-degree) of vertex i, denoted as δ^{in} (respectively, δ^{out}), is the number of edges that enter (respectively, exit) vertex i. Since each vertex has an individual in- and out-degree, it is possible to compute the average and the variance of this collection of numbers.

The number of edges is the cardinality of the edge set; the numbers of positive and negative edges are

$$\#P = \sum_{i,j \in \mathcal{V} : w_{ij} > 0} 1 \qquad \text{and} \qquad \#N = \sum_{i,j \in \mathcal{V} : w_{ij} < 0} 1. \tag{8}$$

The bidirectional coefficient is computed as the ratio between the number of bidirectional edges (an edge connecting the same vertices exists in both directions) and the total number of edges (excluding self-loops):

$$Bc = \frac{\sum_{i \neq j \in \mathcal{V}} |w_{ij} w_{ji}|}{\sum_{i \neq j \in \mathcal{V}} |w_{ij}|}. \tag{9}$$

Finally, the positive to negative edge ratio corresponds to the ratio between the number of positive edges and the number of negative edges: $\#P/\#N$.

4 Agreement Plot analysis of additional models

Due to space limitations, the Main Paper only presents the results of the Agreement Plot analysis applied to the Friedkin-Johnsen model and the Classification-based model. However, to show how the proposed methodology can be applied to other agent-based opinion formation models, here we present the analysis results for two more models: the Bounded-Confidence model [3] and the Backfire Effect and Biased Assimilation model [10].

Colour-coding: As explained in the Main Paper, in order to maximise the information obtained by the different plots, the plotted curves and points are colour-coded. Unless otherwise specified, the colour-coding represents the *average agent parameters* of a population. For the Backfire Effect and Biased Assimilation (BEBA) model and for the Bounded Confidence (BC) model, each agent has a single parameter, which is represented by a real number belonging to a given interval. For the BEBA model, the *entrenchment* κ_i of an agent i lies in the interval $(0, 7)$. For the BC model, the *confidence radius* r_i of an agent i lies in the interval $[0, 2]$. Thus, the agent parameters of a population of N individuals are simply a set of N numbers belonging to the corresponding interval. The average of these values yields the average parameter for the whole population. Each set of agent parameters has a single average, which can be encoded in the curves or points representing that population using colour. For the Backfire Effect and Biased Assimilation model, a smaller average entrenchment $\overline{\kappa}$ is associated with green and a higher entrenchment with orange. For the Bounded Confidence model, a smaller average confidence radius $\overline{r}$ (less open-minded population) is associated with lavender and a larger average confidence radius (more open-minded populations) with pink. The colour-coding also extends to the histograms of the agent parameters.

For the BEBA model, the **UDTE** and **UDSS** plots have only 9 lines or points, because – in view of the nature of the model – the digraphs change at every time step. The topology remains the same, but the weights change, and hence the 9 digraphs correspond to the different topologies presented in the main paper. The **UDTE** and **UDSS** plots are not meaningful to consider for the BC model, since both the digraph weights and topology change at every time step.

4.1 Backfire Effect and Biased Assimilation model

We adopt our method to assess the opinion evolution generated by the Backfire Effect and Biased Assimilation model [10], for several different choices of the agent parameters (entrenchment, see Fig 2 for some examples), four different underlying digraph topologies (whose metrics are reported in Table 2 in the Main Paper) and several different initial conditions.

The colour of the curves and points describing the evolution of the BEBA model encode the *average entrenchment* $\overline{\kappa}$. Although originally $\kappa_i > 0$, for the purpose of our analysis we restricted the entrenchment values to $\kappa_i \in (0, 7)$ for all agents. Small values of the average entrenchment are associated with green and generally represent increased openness towards different opinions. Higher values of the average entrenchment are associated with orange and correspond to agents that react negatively to even slightly different opinions, and that are positively influenced only by agents with very similar views.

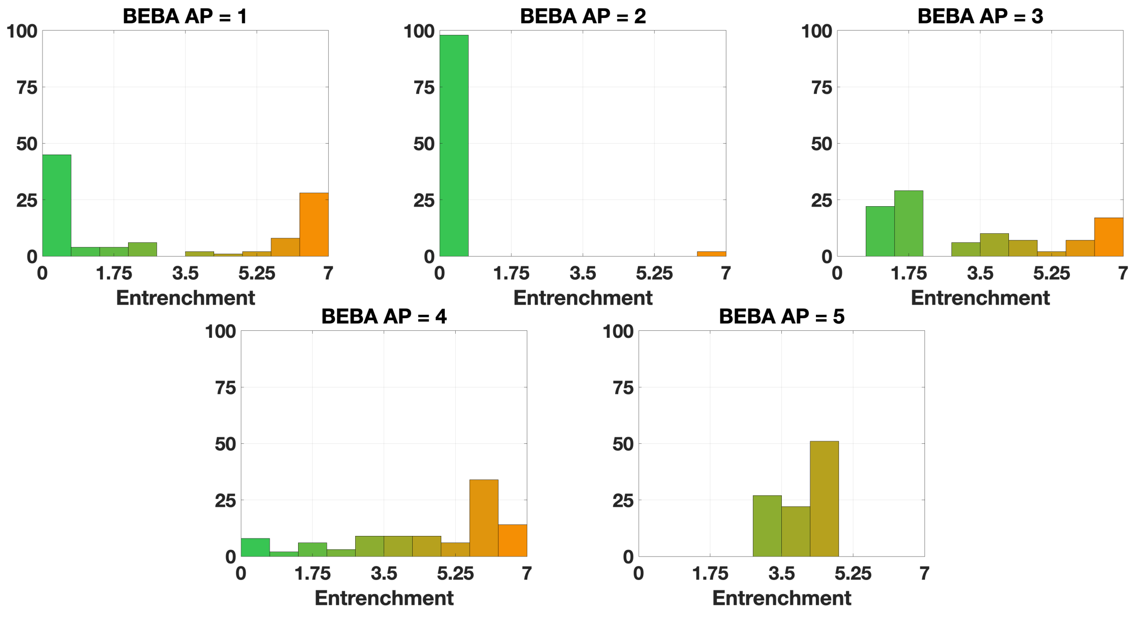

Figure 2: Histograms of different agent parameter choices for the BEBA model with $N = 100$ agents. The height of each bin indicates the number of agents whose entrenchment parameter lies within the considered interval.

Table 1 shows 12 different **APTE** plots, for three different choices of the initial opinion distribution (purple circles)

and four different underlying digraphs. The plots reveal a behaviour unseen in the Friedkin-Johnsen model. For the initial opinion distributions in the second and third row, the average of the opinion absolute values is approximately 0.5 and several parametric curves move to the right. The curves moving to the right have a relevant orange component, and thus correspond to a highly entrenched population, where biased assimilation and backfire effect are predominant, and which are therefore likely to evolve towards regions in the Agreement Plot where the agents have a higher level of interest, to create either polarisation or perfect consensus. On the other hand, curves with a relevant green component (low entrenchment) tend to move to the left, towards the point $(0, 0)$. This behaviour is similar to that of the French-DeGroot model, and indeed the BEBA model with $\kappa \to 0$ is known to behave like the French-DeGroot model. Moreover, as seen with the Friedkin-Johnsen model, the opinions change more slowly with the Scale-Free digraph and faster with the other digraphs.

Table 2 shows 12 different **UDTE** plots, for three different initial opinions and 4 different choices of the agent parameters, and suggests two observations. First, besides the opinion change speed, different digraph topologies do not dramatically affect the model evolution. Some sporadic examples where not all lines follow the same direction can be seen in row 2 and 3, and column 2. Nevertheless, most curves appear to follow the same trajectory. Second, the mean entrenchment appears to be a good predictor of the system behaviour, like with the Friedkin-Johnsen model. This can be seen in columns 2, 3, and 4, where the agent parameters were different but had comparable mean (seen in the similarity of the line colours) and the behaviour is qualitatively comparable.

Table 3 shows 12 different **IOTE** plots, for three different choices of the agent parameters (corresponding to the third, first and fourth histograms in Fig 2) and four different underlying digraphs. In all the plots, some curves move to the left and others to the right. Since in these plots the agent parameters and the underlying graph are always the same, the tendency of the opinions to go either to extremes or to consensus depends on the location of the initial opinions in the Agreement Plot. A closer inspection reveals that some plots have a threshold value for $\overline{|x|}$ that separates the curves that move to the left from those that move to the right: see for instance the plot in the third row and fourth column. For other plots, such as the one in the first row and third column, no neat threshold can be identified. Actually, two types of curves move to the right: one type starts moving to the right immediately, while the other moves initially to the left and then turns to the right. A possible explanation for this fact is that, for the first type, the backfire effect is predominant and causes polarisation and seeking of extreme opinions from the start; while for the second type, opinions tend to form consensus and are then reinforced, thus moving towards consensus or perfect consensus at higher levels of interest (large $\overline{|x|}$).

Table 4 shows 12 different **APSS** plots for three different choices of the initial opinion distribution (purple circles) and four different underlying digraphs. These plots clearly show the capacity of the BEBA model to produce a wide variety of final opinion distributions. We can observe that: (i) almost every point along the line $\overline{x} = 1$ is reached, meaning that the model can lead to final opinion distributions where all the opinions are extreme (either -1 or 1) with any proportion (the average ranges from 1 to -1); (ii) not every orange point is located near $(1, 0)$, meaning that very high entrenchment does not automatically lead to polarisation; (iii) the digraph topology has a strong effect on the possible model outcomes and every digraph produces a significantly different plot; (iv) although final opinion distributions can be found in almost every point inside the triangle, the neighbourhood of the point $(0.1, 0)$ is more empty, which suggests that, even when the initial opinion distribution is located in that area, the opinions evolve so that the point moves to the left until it reaches the triangle boundary.

Table 5 shows 12 different **UDSS** plots for three different initial opinions and four different choices of the agent parameters. Plots in Table 5 show that, in most cases, the initial opinion and the agent parameters have a greater effect than the underlying digraph over the location of the predicted opinions: for most plots, the predicted points are closely located. Combining this observation with the observations from Table 4, we can say that different underlying digraphs enable different model predictions, but that these different model predictions are ultimately achieved by the agent parameters themselves.

Table 6 presents 12 different **IOSS** plots showing how the reference initial opinion distributions (in Fig 3B of the Main Paper) evolve for three different choices of the agent parameters (corresponding to the third, first and fourth histograms in Fig 2) and four different underlying digraphs. Interestingly, the plots highlight the rich and diverse behaviour of the BEBA model. The plot in the first row and first column confirms that, with given agent parameters and underlying digraph, the model can produce extreme opinions (in fact, opinions along the lines $\overline{x} = \pm\overline{|x|}$ can be associated with perfect consensus, while opinions along the line $\overline{|x|} = 1$ are associated with either perfect consensus or polarisation). The Scale-Free topology leads to a peculiar model behaviour, apparent in every row, in particular the first and the third. The second row is very intriguing, in particular in the fourth column. The points in the plots of the second row can be separated into two types: points located along the lines $\overline{x} = \pm\overline{|x|}$, for which either all agents agree or all agents disagree; points forming an 'arc' that is more or less defined depending on the underlying digraph. A similar arc was formed with the Friedkin-Johnsen model; also in this case, the opinions tend to contract and move towards consensus. Considering the agent parameters for the second row allows us to give a deeper meaning to the two types of points: as shown in the agent parameter histogram, some agents have a very low entrenchment, while others have a very high entrenchment. Hence, depending on where the initial opinion distributions are located in the $(0, 0)$, $(1, -1)$, $(1, 1)$ triangle, one of the two groups of agents has a predominant effect. When the initial opinions are already polarised, the backfire effect has nowhere to move the opinions, and thus the predominant effect is due

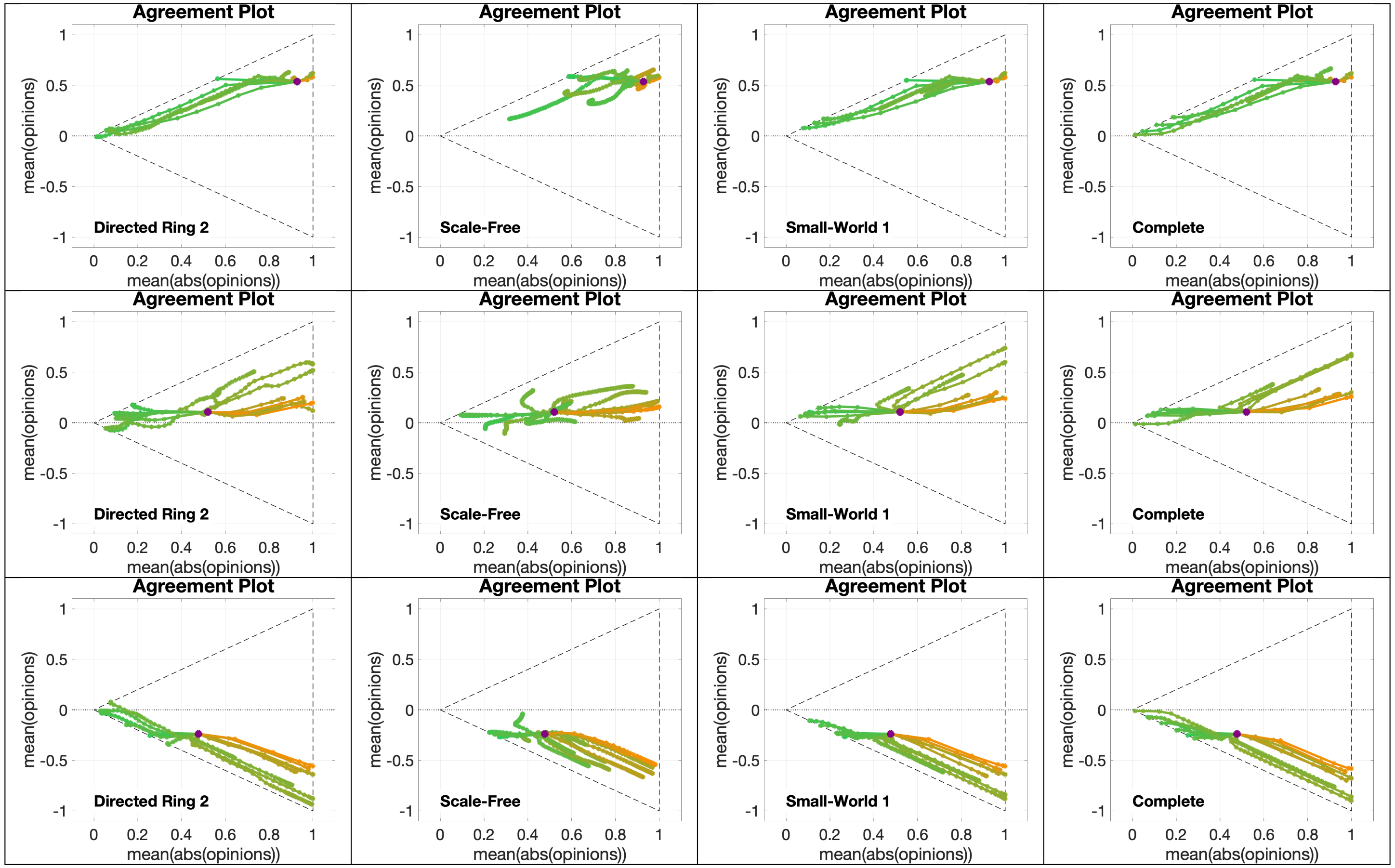

Table 1: **APTE** plots for the Backfire Effect and Biased Assimilation model: each of the 12 plots includes 15 curves associated with different choices of the agent parameters, all with the same initial opinion distribution and underlying digraph. Plots in the same row have the same initial opinion distribution (purple circle). Plots in the same column have the same underlying digraph (from left to right, Directed Ring, Scale-Free, Small-World, Complete). All the simulations were performed for 50 time steps and 100 agents.

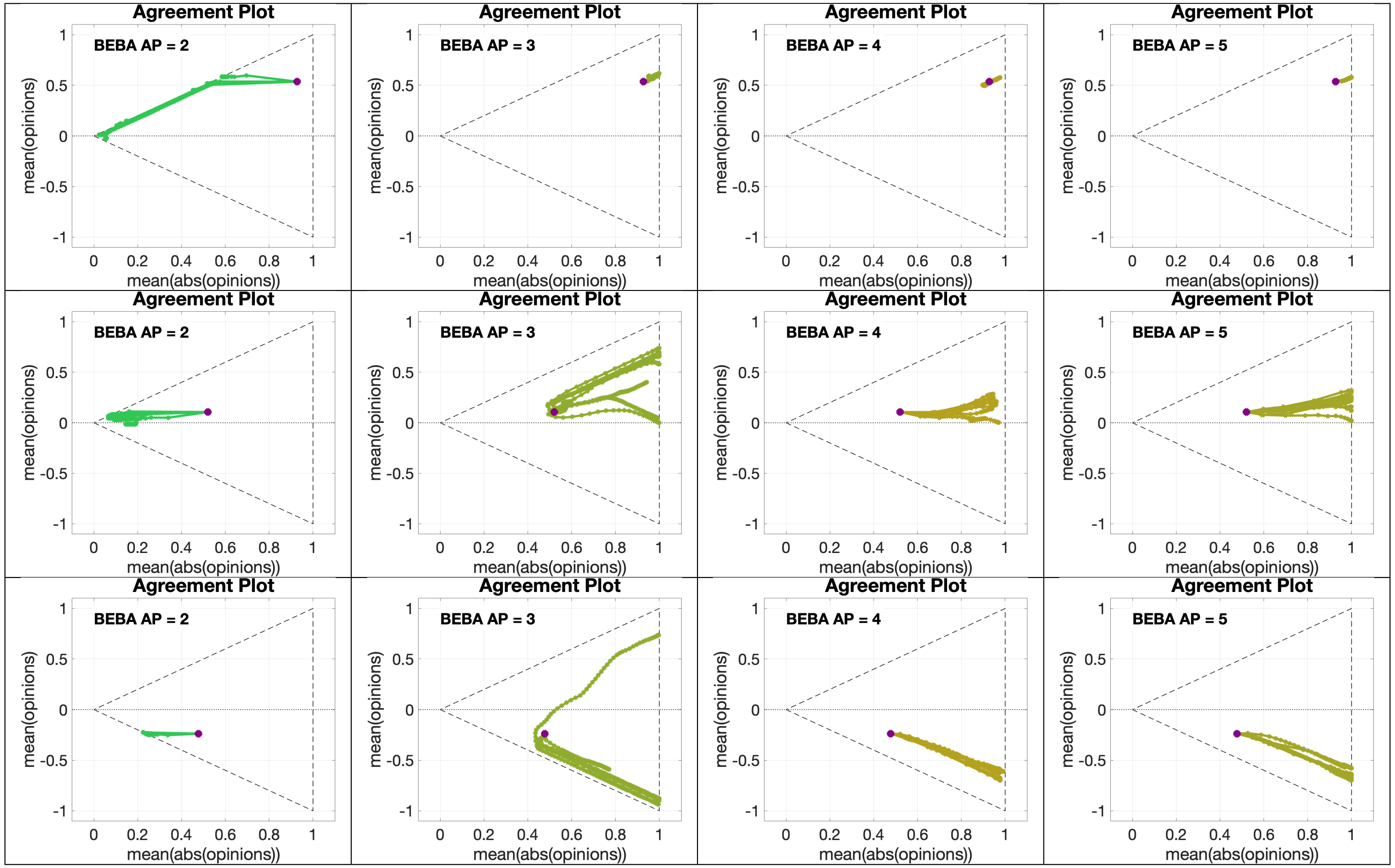

Table 2: **UDTE** plots for the Backfire Effect and Biased Assimilation model: each of the 12 plots includes 9 curves with different choices of the underlying digraphs, all with the same initial opinion distributions and agent parameters. Plots in the same row have the same initial opinion distribution (purple circle). Plots in the same column have the same agent parameters. All the simulations were performed for 50 time steps and 100 agents.

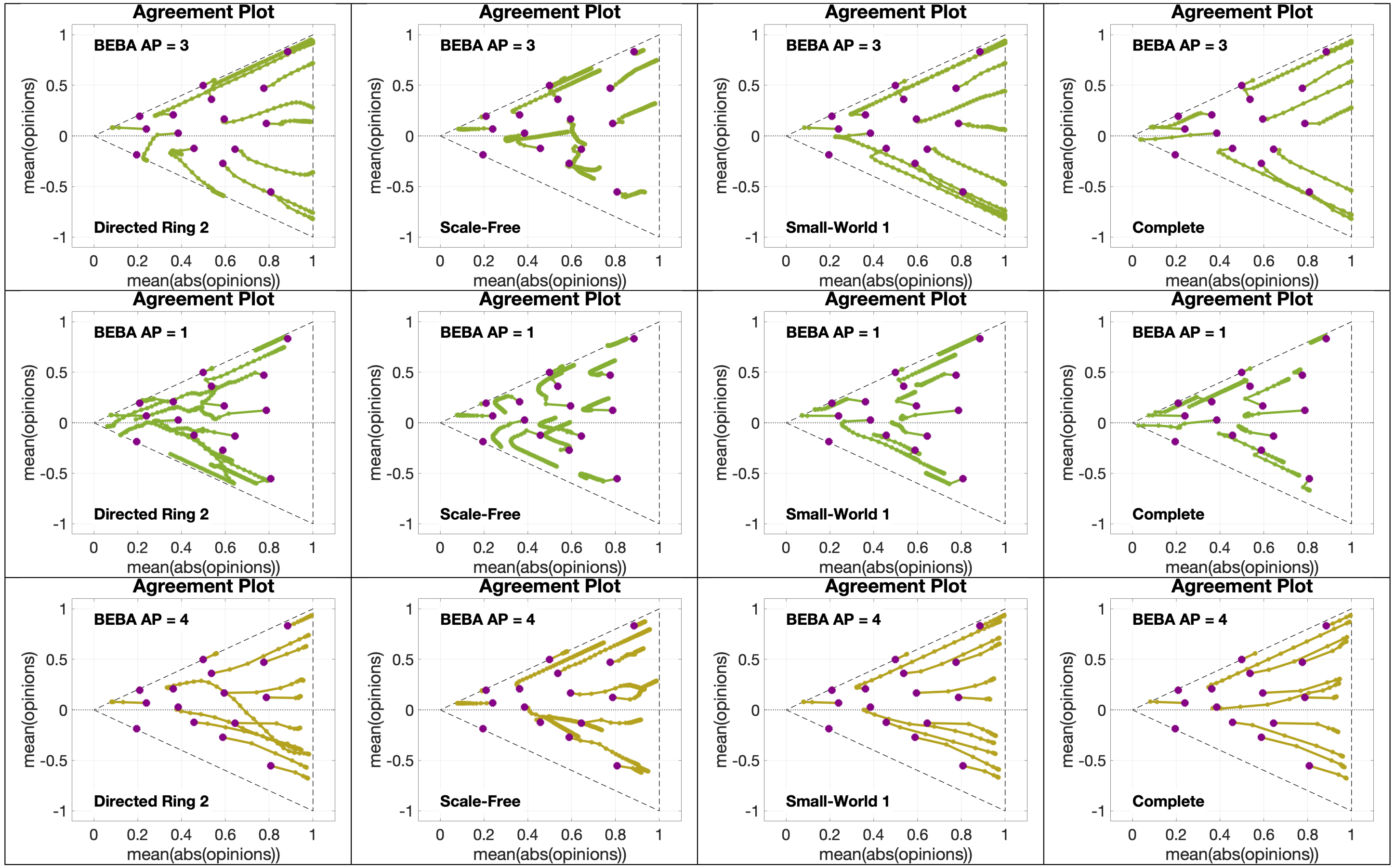

Table 3: **IOTE** plots for the Backfire Effect and Biased Assimilation model: each of the 12 plots includes 15 curves associated with different choices of the initial opinion distributions, all with the same agent parameters and underlying digraph. Plots in the same row have the same agent parameters (associated with the third, first and fourth histograms in Fig 2). Plots in the same column have the same underlying digraph (from left to right, Directed Ring, Scale-Free, Small-World, Complete). All the simulations were performed for 50 time steps and 100 agents.

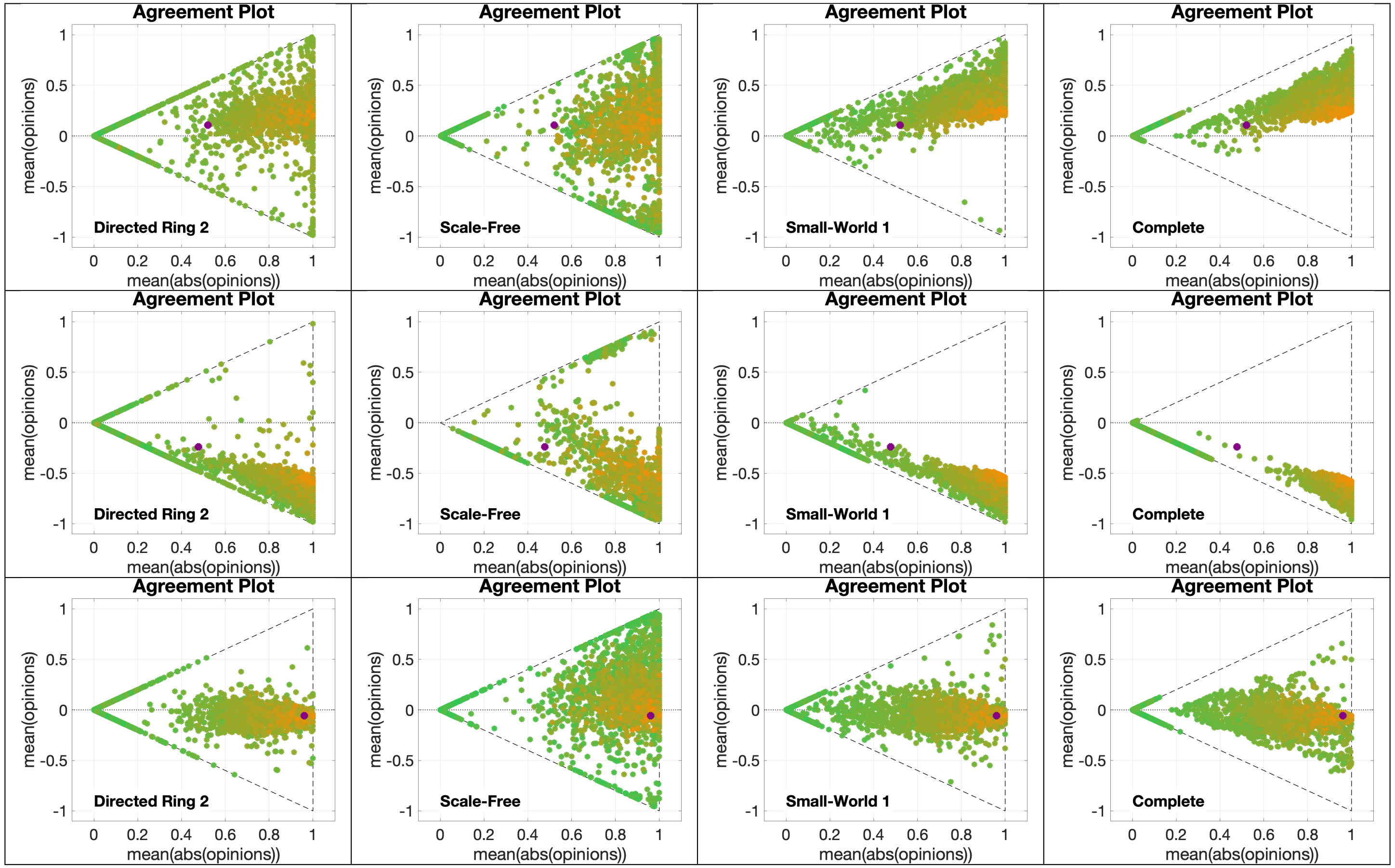

Table 4: **APSS** plots for the Backfire Effect and Biased Assimilation model: each of the 12 plots includes 3528 points associated with different choices of the agent parameters, all with the same initial opinion distribution and underlying digraph. Plots in the same row have the same initial opinion distribution (purple circle). Plots in the same column have the same underlying digraph (from left to right, Directed Ring, Scale-Free, Small-World, Complete). All the simulations were performed for 1000 time steps and 100 agents.

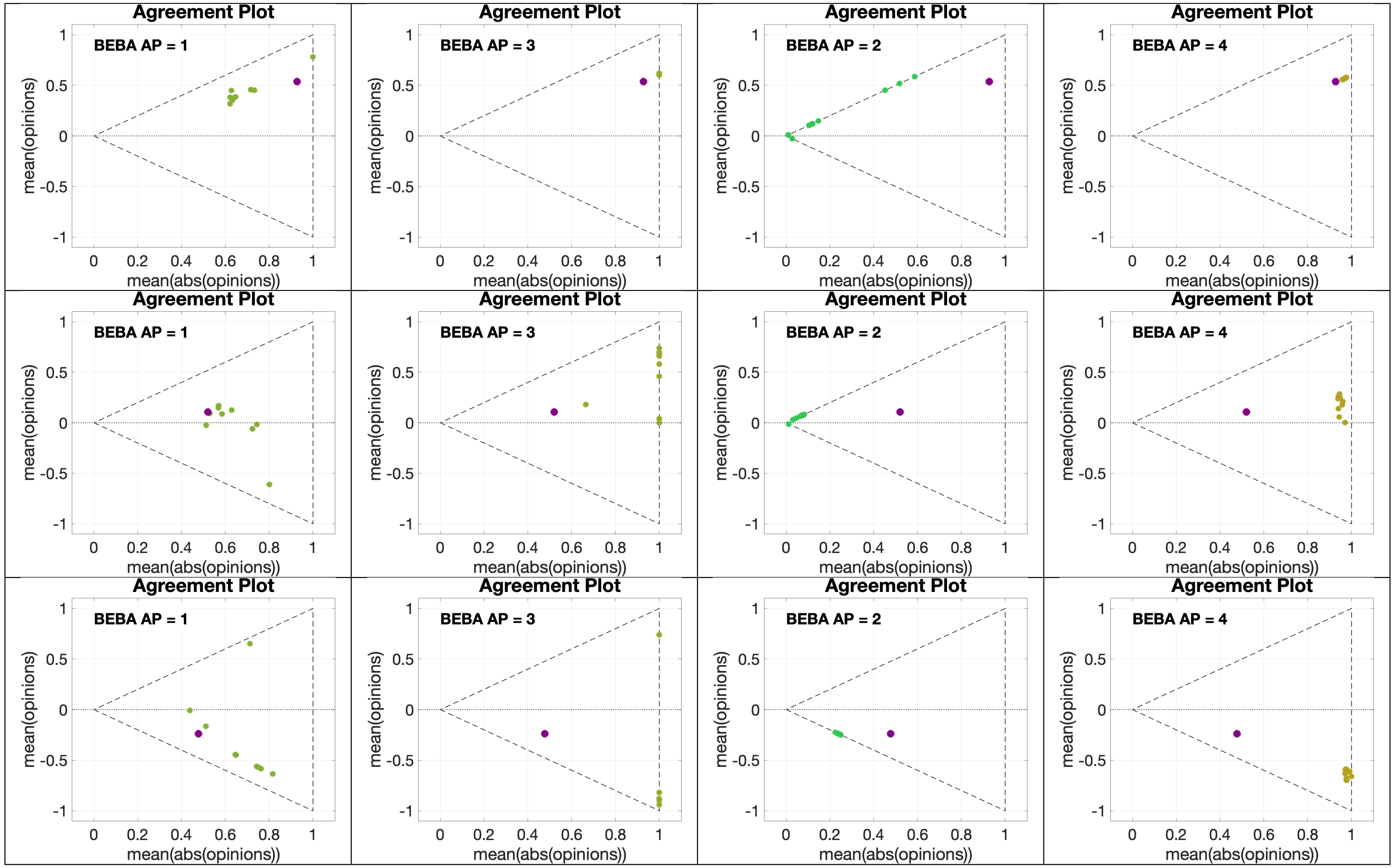

Table 5: **UDSS** plots for the Backfire Effect and Biased Assimilation model: each of the 12 plots includes 9 points associated with different choices of underlying digraphs, all with the same initial opinion distributions and agent parameters. Plots in the same row have the same initial opinion distribution (purple circle). Plots in the same column have the same agent parameters. All the simulations were performed for 1000 time steps and 100 agents.

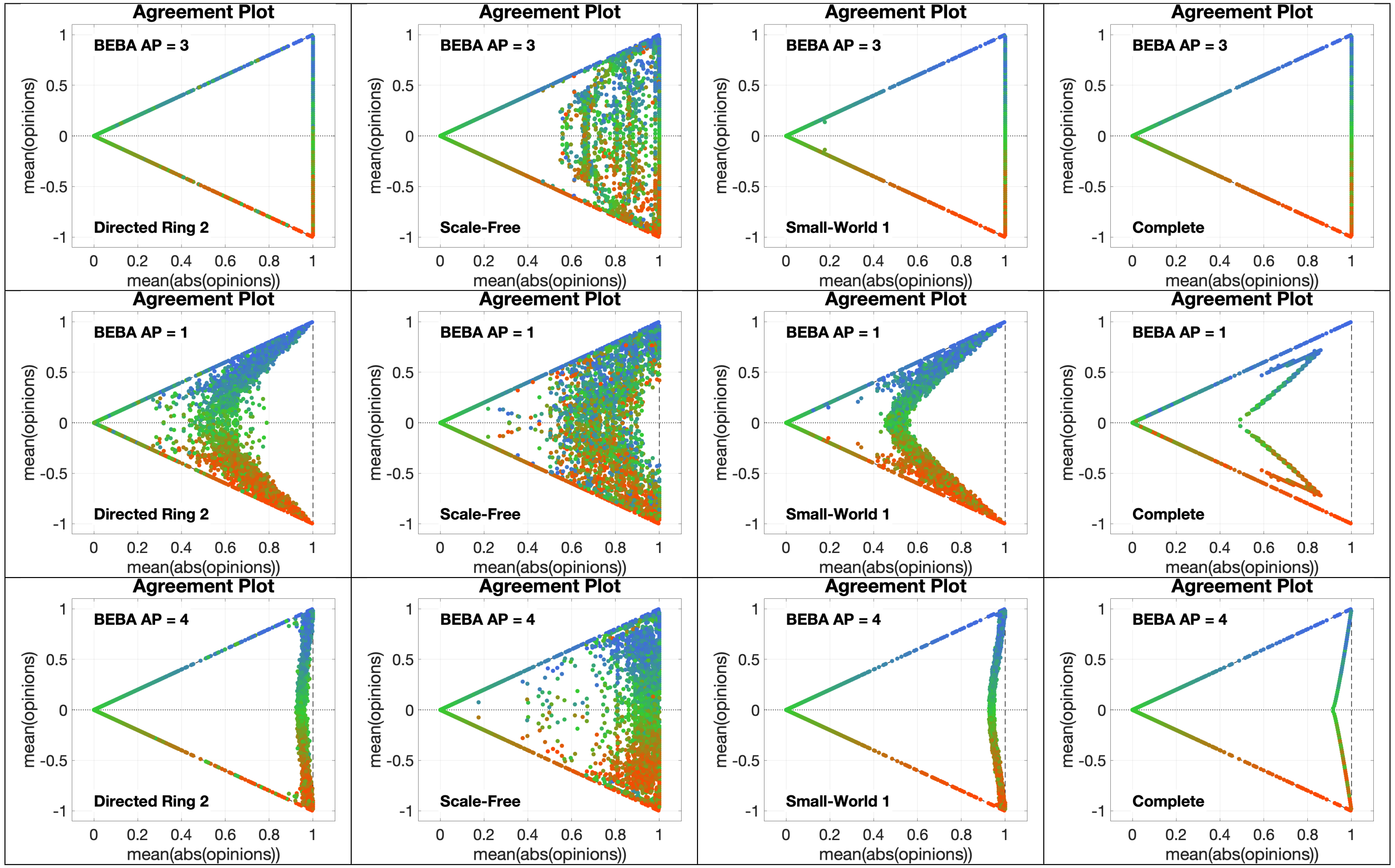

Table 6: **IOSS** plots for the Backfire Effect and Biased Assimilation model: each of the 12 plots includes 5314 points associated with different choices of the initial opinion distributions, all with the same agent parameters and underlying digraph. Plots in the same row have the same agent parameters. Plots in the same column have the same underlying digraph (from left to right, Directed Ring, Scale-Free, Small-World, and Complete). All the simulations were performed for 1000 time steps and 100 agents.

to less entrenched agents that slightly move the opinion distribution towards consensus. On the other hand, when the initial opinions are near consensus or perfect consensus, the biased assimilation effect of the highly entrenched agents is predominant and moves the opinion distribution towards consensus or perfect consensus, i.e. towards the lines $\overline{x} = \pm\overline{|x|}$.

Our graphical analysis casts light onto the opinion evolution capabilities of the BEBA model for a variety of initial opinion distributions and agent parameters: the model exhibits a rich and varied behaviour, resulting from the inclusion of both the backfire effect and the biased assimilation mechanism in the opinion formation process. In particular, the backfire effect allows for opinion distributions to achieve extreme opinion values, resulting in perfect consensus and polarisation. On the other hand, the biased assimilation mechanism moves opinions towards consensus on opinions that can have any value in the interval $[-1, 1]$.

4.2 Bounded Confidence model

We now adopt our method to assess the opinion evolution generated by the Bounded Confidence model [3], for several different choices of the agent parameters (confidence radius, see Fig 3 for some examples) and several different initial conditions. For the Bounded Confidence model, the digraph topology changes at each time step, therefore there is no constant underlying digraph, while the agent parameter is the confidence radius $r_i \in [0, 2]$: a confidence radius near 0 is associated with a lavender colour, while a confidence radius near 2 is associated with a pink colour.

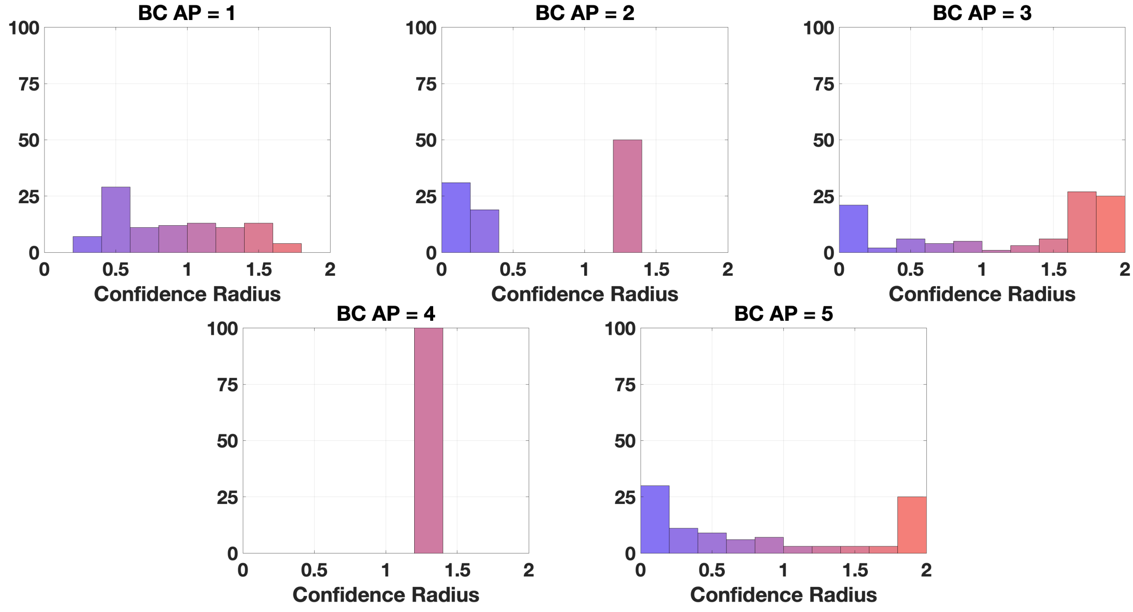

Figure 3: Histograms of different agent parameter choices for the Bounded Confidence model with $N = 100$ agents. The height of each bin indicates the number of agents whose confidence radius lies within the considered interval.

Table 7 presents the **APTE** plots for four different choices of the initial opinion distribution (orange circles). As with the Friedkin-Johnsen model, all the parametric curves move to the left, meaning that the opinions become less extreme: the Bounded Confidence model has no mechanism to move opinions away from each other, and therefore new opinions cannot exceed the largest and smallest initial opinions. In contrast to the Friedkin-Johnsen model, the parametric curves also have a significant component along the vertical axis. A possible explanation is the following: if there are two subgroups of agents with distant opinions, one with agents having large confidence radius and the other with agents having small confidence radius, the first group will be influenced by the second group, but not vice-versa, resulting in a change in the average of the opinions.

Table 8 shows the **IOTE** plots for four different choices of the agent parameters. The resulting behaviour is to be expected of a model that, in essence, averages opinions: the parametric curves move to the left. However, in some plots, some points seem not to have an actual evolution: in fact, for those initial opinion distributions, the confidence radii of the agents are such that there is no influence among the agents (or, if there is, it is very small and therefore not noticeable). Indeed, the points without a visible trajectory are located to the right of the plots, where the average of the absolute values of the opinions is relatively large, meaning that most agents have extreme (and hence, distant) opinions.

Table 9 presents the **APSS** plots for four different initial opinion distributions. They are similar to the ones obtained for the Friedkin-Johnsen model, with a key difference: some steady-state points can be located to the right of the initial opinion distribution point, and not near the lines $\overline{x} = \pm\overline{|x|}$. Although not a common outcome, it is also significant. The same explanation as before applies: one subgroup of agents with extreme opinions can influence

the other agents without being influenced in turn, and thus move them to more extreme opinions without necessarily achieving consensus. Most of the points located to the right of the initial opinion point and not near the lines $\overline{x} = \pm\overline{|x|}$ are lavender (small average confidence radius); therefore, if only some agents have a relatively large confidence radius, they will move towards the other agents, which could possibly have a more extreme opinion. Also, most of the lavender points are close to the initial opinion distribution: in fact, if the average confidence radius is small, then very few edges will be formed to connect the agents and the opinions will not change significantly. On the other hand, pink points tend to be near the lines $\overline{x} = \pm\overline{|x|}$: when the conference radius is large, the opinion evolution is similar to that generated by the French-DeGroot model and thus yields perfect consensus.

Table 10 shows the **IOSS** plots for four different choices of the agent parameters. The points in these plots can be divided into two types: a contracting type and an invariant type. The contracting type moves towards the left (consensus), but the average of the opinions can change significantly: some azure points have a negative $\overline{x}$, while some orange points have a positive $\overline{x}$. This is consistent with the fact that parametric curves have a significant component along the vertical axis. The invariant type is characterised by initial opinion distributions where the agents do not (or only slightly) influence one another, because their confidence radius is smaller than the opinion differences, and therefore the opinions remain almost the same. These explanations are further backed by observing the agent parameters in each plot. In the first plot, some agent parameters can have a confidence radius of 2 and therefore are influenced by all the other agents and tend to consensus (which can influence other agents with narrower confidence radius to also move), resulting in a strong contraction and in the absence of points of the invariant type. In the third plot, the maximum confidence radius is around 1.9, and therefore opinions with an absolute value average above 0.95 tend to remain invariant (since individual opinions are either close together or farther than a distance of 1.9), while the other opinion distributions contract. For the second and fourth plots, the maximum confidence radius is even smaller, resulting in even more initial points of the invariant type. Unlike in the other plots, for the points of the contracting type in the fourth plot, the opinions are exactly on the lines $\overline{x} = \pm\overline{|x|}$, because all agents have very similar confidence radius and therefore the resulting digraphs are Complete and all the agents converge to the same opinion. This does not happen in the other plots, where some agents have a very small confidence radius and therefore their opinion is almost invariant, preventing the steady state opinions from forming perfect consensus.

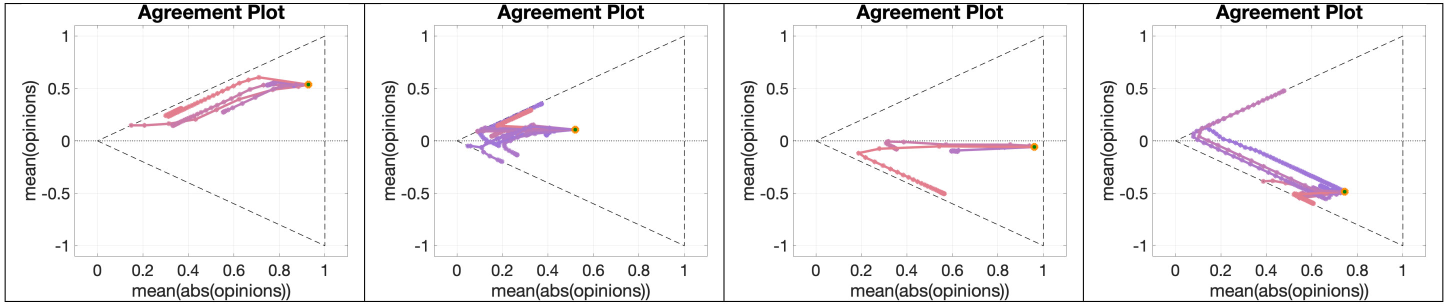

Table 7: **APTE** plots for the Bounded Confidence model: each of the 4 plots includes 15 curves associated with different choices of the agent parameters, all with the same initial opinion distribution (orange circle). Each plot corresponds to a different initial opinion distribution. All the simulations were performed for 50 time steps and 100 agents.

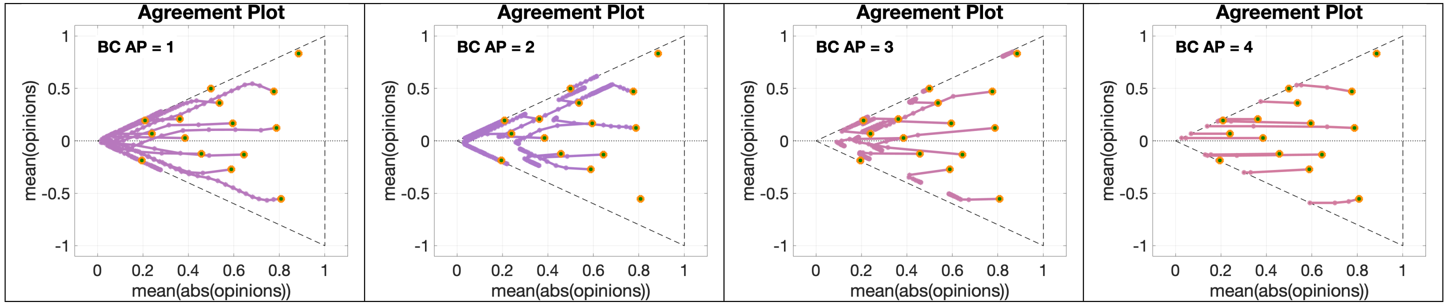

Table 8: **IOTE** plots for the Bounded Confidence model: each of the 4 plots includes 15 curves associated with different choices of the initial opinion distributions, all with the same agent parameters. Each plot corresponds to a different choice of the agent parameters (associated with the first, second, third or fourth histogram in Fig 3). All the simulations were performed for 50 time steps and 100 agents.

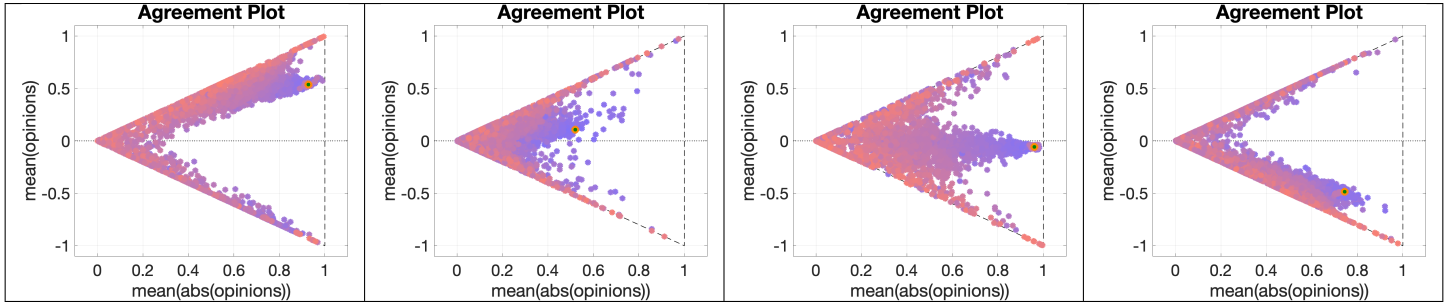

Table 9: **APSS** plots for the Bounded Confidence model: each of the 4 plots includes 3528 points associated with different choices of the agent parameters, all with the same initial opinion distribution. Each plot corresponds to a different initial opinion distribution (orange circle). All the simulations were performed for 1000 time steps and 100 agents.

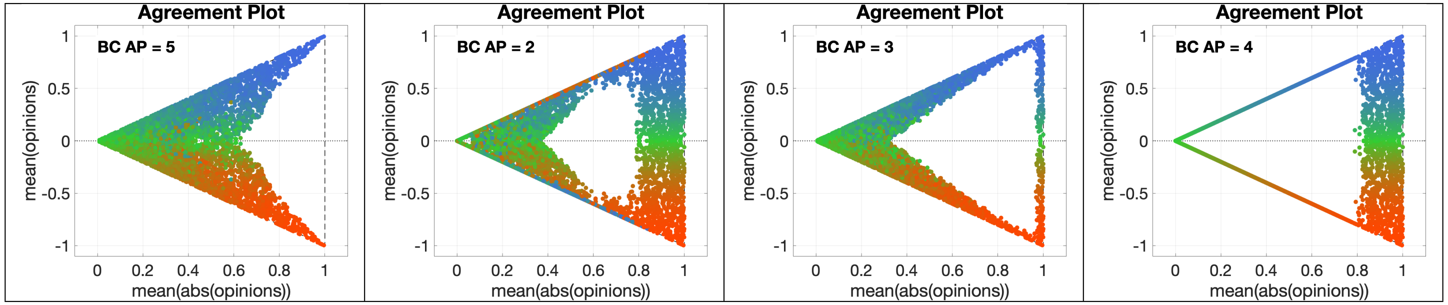

Table 10: **IOSS** plots for the Bounded Confidence model: each of the 4 plots includes 5314 points associated with different choices of the initial opinion distributions, all with the same agent parameters. Each plot corresponds to a different choice of the agent parameters (associated with the fifth, second, third or fourth histogram in Fig 3). All the simulations were performed for 1000 time steps and 100 agents.

References

[1] Sandrock C. alchemyst/ternplot; 2012. Available at `https://www.mathworks.com/matlabcentral/fileexchange/2299-alchemyst-ternplot?s_tid=srchtitle` (2022/06/13).

[2] Devia CA, Giordano G. Classification-Based Opinion Formation Model Embedding Agents' Psychological Traits. Journal of artificial societies and social simulation. 2023.

[3] Hegselmann R, Krause U. Opinion dynamics and bounded confidence models, analysis, and simulation. Journal of artificial societies and social simulation. 2002;5(3).

[4] French Jr J. A formal theory of social power. Psychological Review. 1956.

[5] Harary F. A criterion for unanimity in French's theory of social power. Studies in social power. 1959.

[6] Harary F, Harary A, Norman RZ, Cartwright D, Esau K. Structural Models: An Introduction to the Theory of Directed Graphs. vol. 82. Wiley; 1965.

[7] DeGroot M. Reaching a consensus. Journal of the American Statistical Association. 1974.

[8] Friedkin N. A formal theory of social power. Journal of Mathematical Sociology. 1986.

[9] Friedkin N, Johnsen E. Social Influence Networks and Opinion Change. Advances in Group Processes. 1999;16.

[10] Chen X, Tsaparas P, Lijffijt J, De Bie T. Opinion dynamics with backfire effect and biased assimilation. PloS ONE. 2021;16(9):e0256922.

[11] Watts DJ, Strogatz SH. Collective dynamics of 'small-world' networks. Nature. 1998;393(6684):440–442.

A High-resolution images for figures in the Main Paper

A.1 Friedkin-Johnsen model

In this appendix, we provide higher resolution versions for the figures in the Main Paper related to the graphical analysis of the Friedkin-Johnsen model.

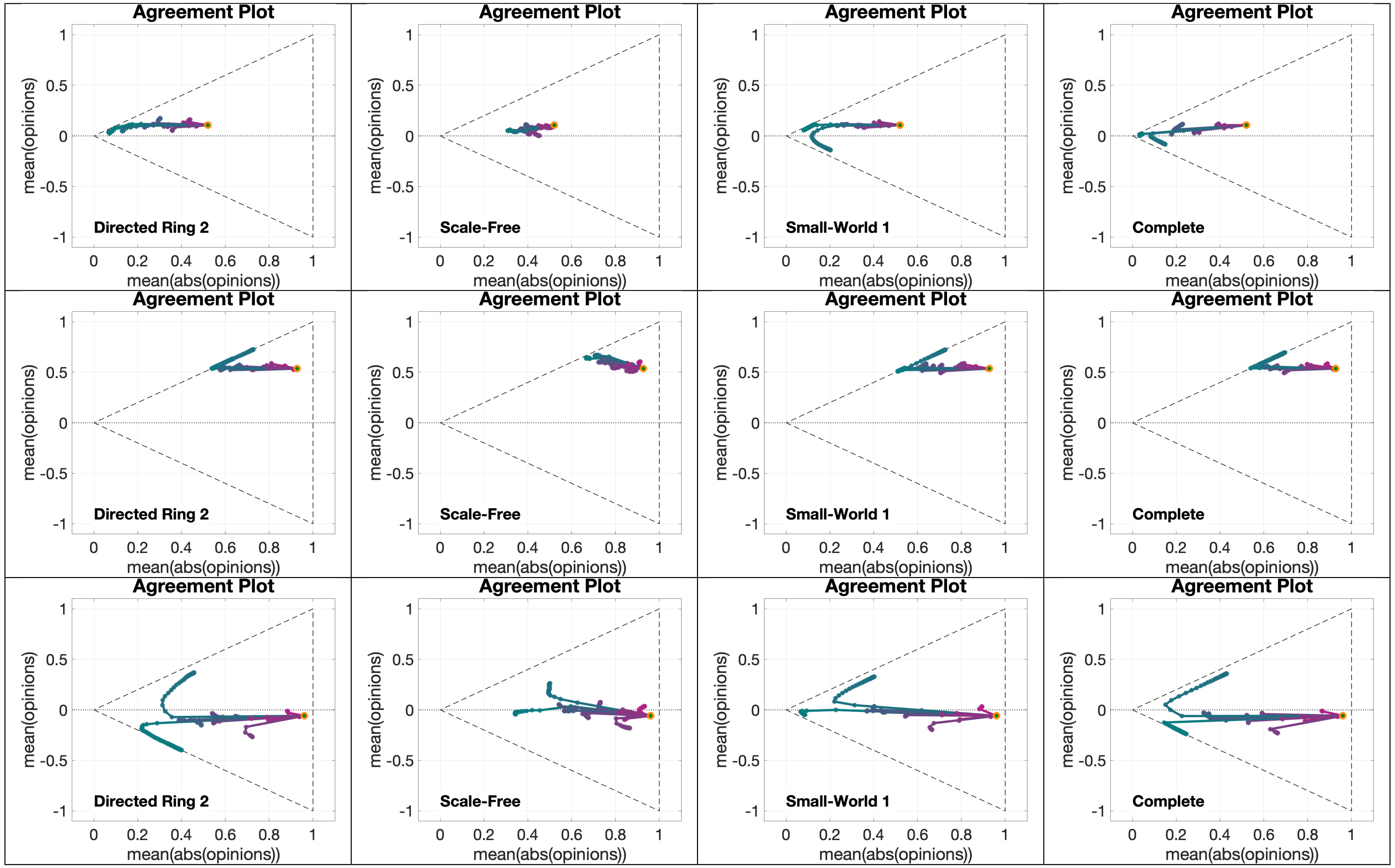

Table 11: **APTE** plots for the Friedkin-Johnsen model: each of the 12 plots includes 15 curves associated with different choices of the agent parameters, all with the same initial opinion distribution and underlying digraph. Plots in the same row have the same initial opinion distribution (orange circle). Plots in the same column have the same underlying digraph (from left to right, Directed Ring, Scale-Free, Small-World, Complete). All the simulations were performed for 50 time steps and 100 agents.

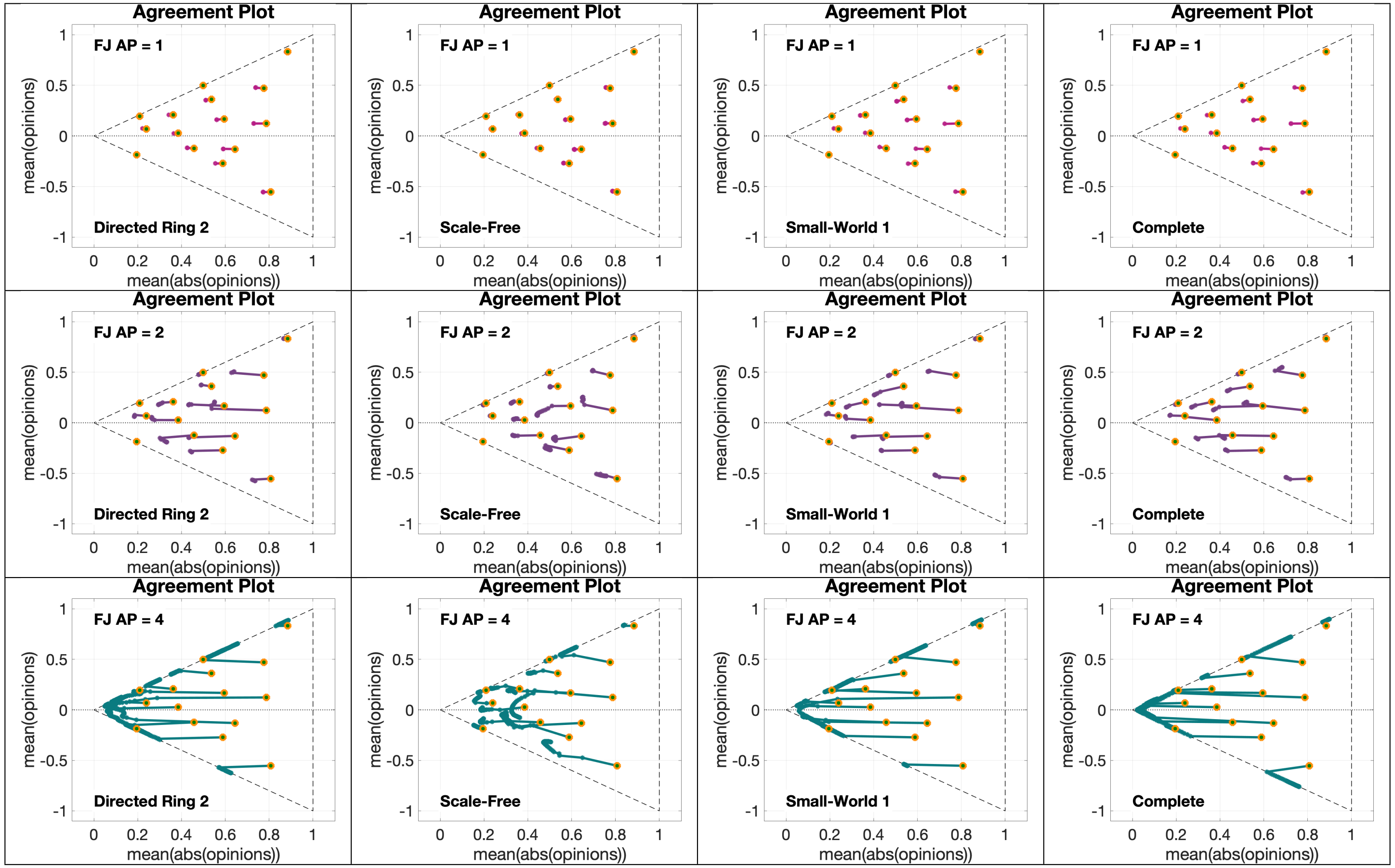

Table 12: **IOTE** plots for the Friedkin-Johnsen model: each of the 12 plots includes 15 curves associated with different choices of the initial opinion distributions, all with the same agent parameters and underlying digraph. Plots in the same row have the same agent parameters (associated with the first, second and fourth histograms in Fig 4 of the Main Paper). Plots in the same column have the same underlying digraph (from left to right, Directed Ring, Scale-Free, Small-World, Complete). All the simulations were performed for 50 time steps and 100 agents.

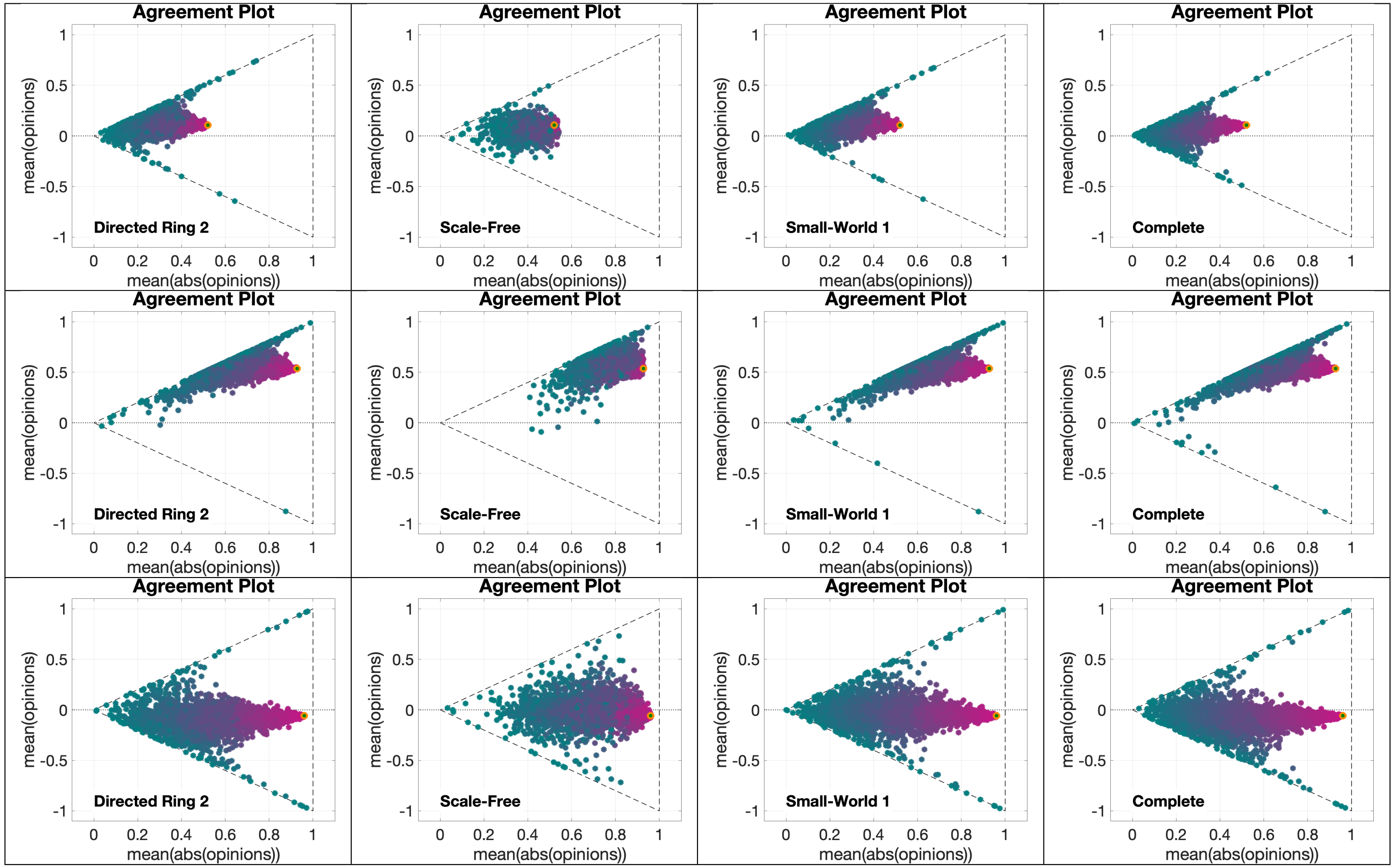

Table 13: **APSS** plots for the Friedkin-Johnsen model: each of the 12 plots includes 3528 points associated with different choices of the agent parameters, all with the same initial opinion distribution and underlying digraph. Plots in the same row have the same initial opinion distribution (orange circle). Plots in the same column have the same underlying digraph (from left to right, Directed Ring, Scale-Free, Small-World, Complete). All the simulations were performed for 1000 time steps and 100 agents.

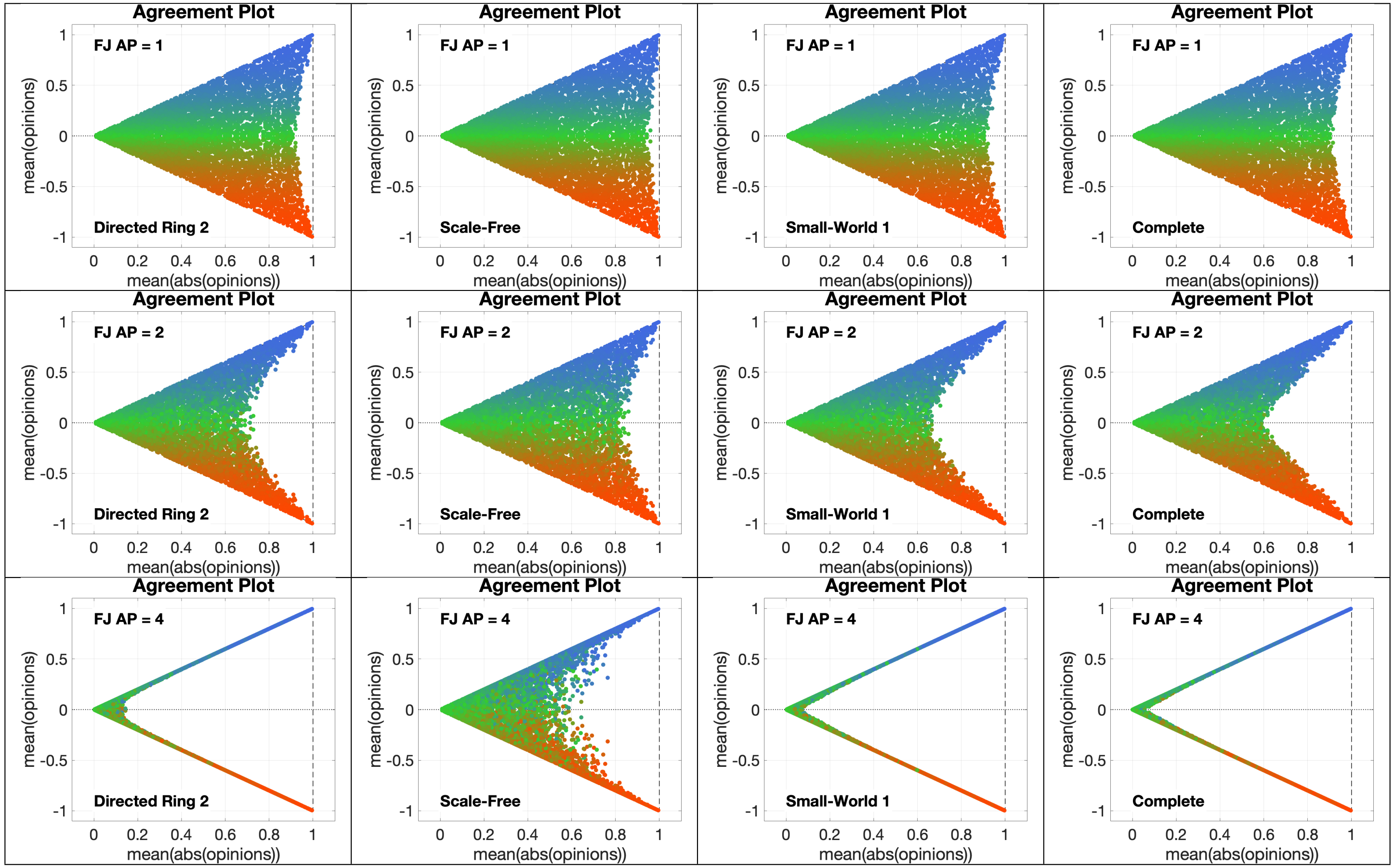

Table 14: **IOSS** plots for the Friedkin-Johnsen model: each of the 12 plots includes 5314 points associated with different choices of the initial opinion distributions, all with the same agent parameters and underlying digraph. Plots in the same row have the same agent parameters. Plots in the same column have the same underlying digraph (from left to right, Directed Ring, Scale-Free, Small-World, and Complete). All the simulations were performed for 1000 time steps and 100 agents.

A.2 Classification-based model

In this appendix, we provide higher resolution versions for the figures in the Main Paper related to the graphical analysis of the Classification-based model.

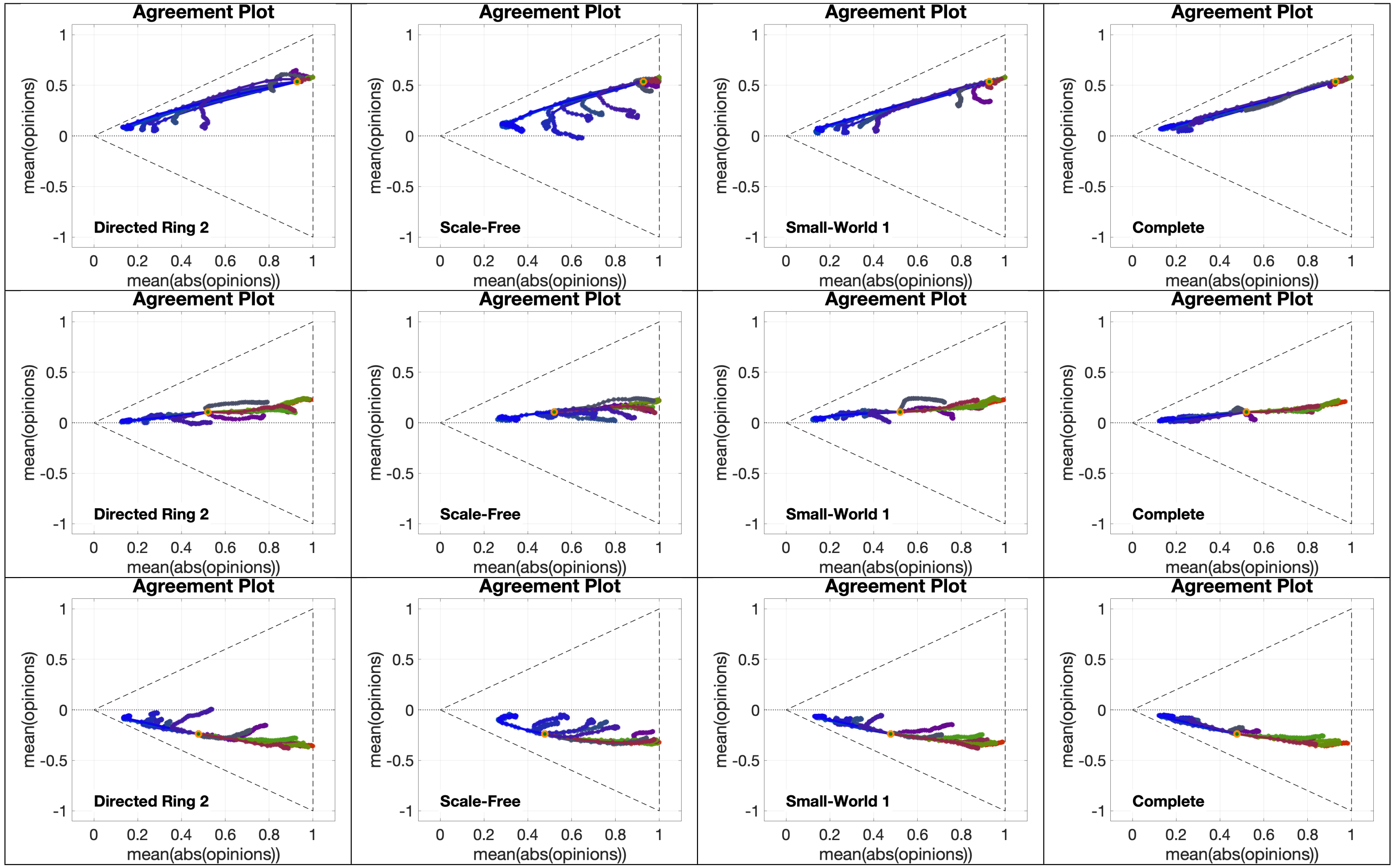

Table 15: **APTE** plots for the Classification-based model: each of the 12 plots includes 15 curves associated with different choices of the agent parameters, all with the same initial opinion distribution and underlying digraph. Plots in the same row have the same initial opinion distribution (orange circle). Plots in the same column have the same underlying digraph (from left to right, Directed Ring, Scale-Free, Small-World, Complete). All the simulations were performed for 50 time steps and 100 agents.

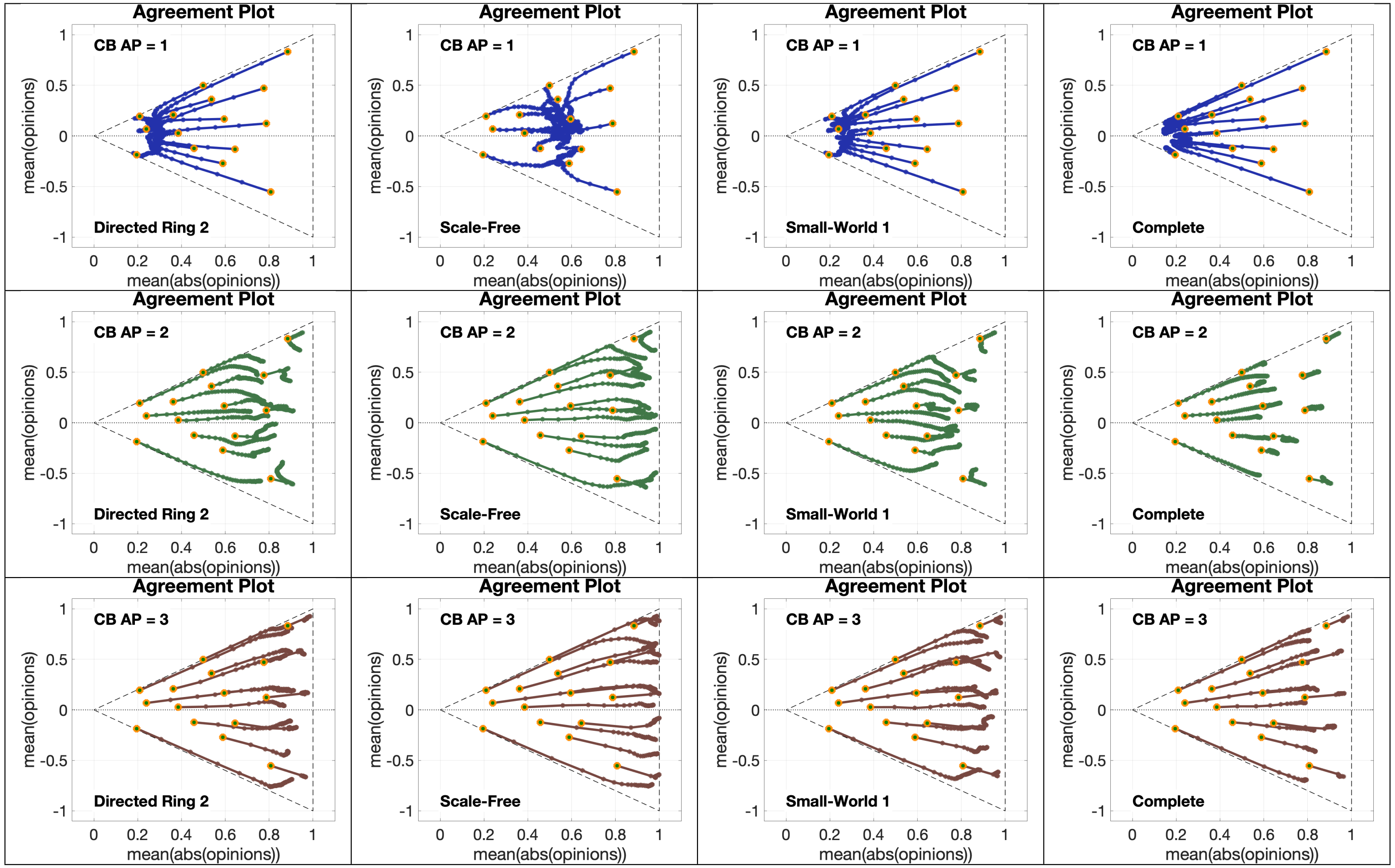

Table 16: **IOTE** plots for the Classification-based model: each of the 12 plots includes 15 curves associated with different choices of the initial opinion distributions, all with the same agent parameters and underlying digraph. Plots in the same row have the same agent parameters (associated with the first three ternary diagrams in Fig 9 of the Main Paper). Plots in the same column have the same underlying digraph (from left to right, Directed Ring, Scale-Free, Small-World, Complete). All the simulations were performed for 50 time steps and 100 agents.

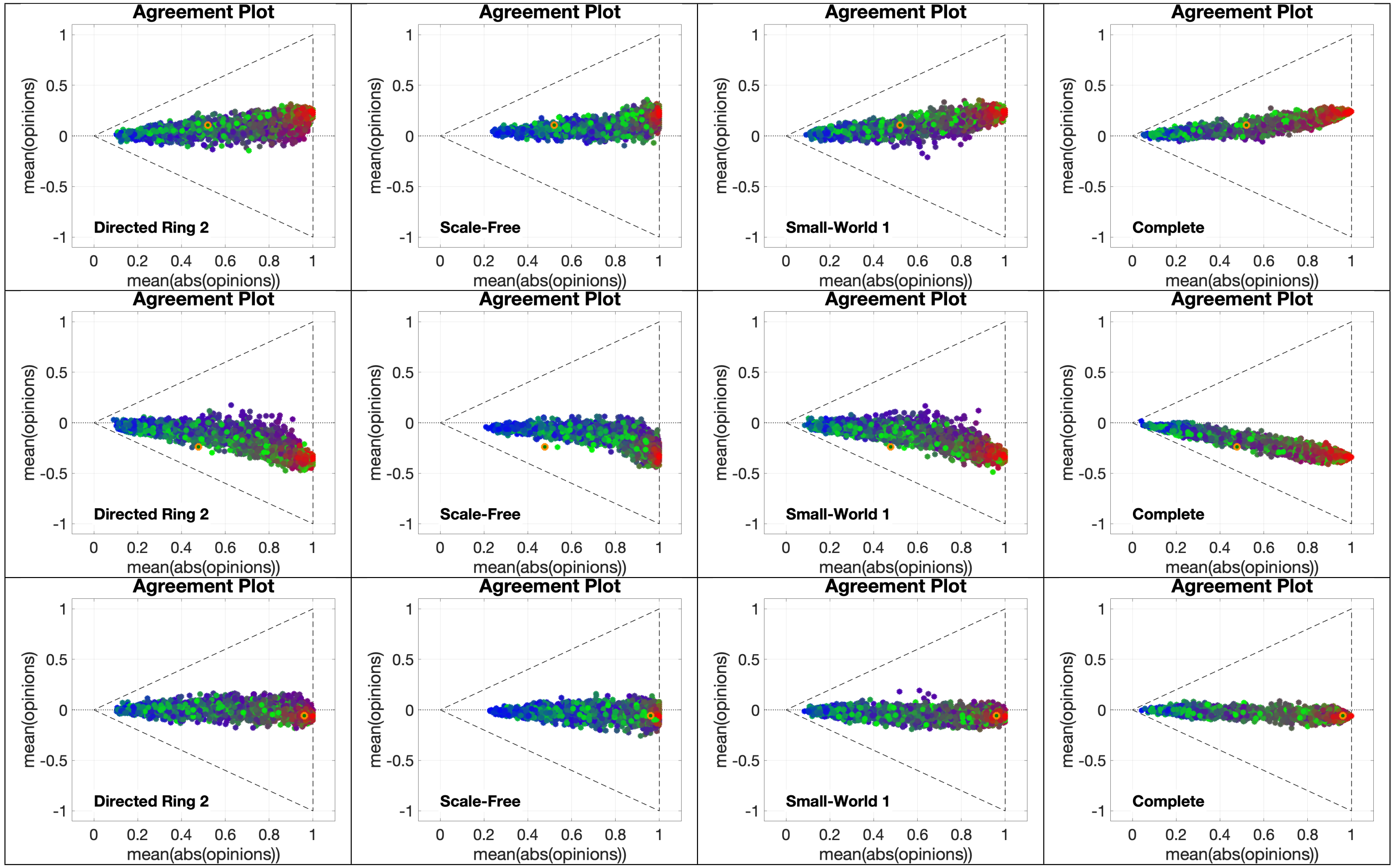

Table 17: **APSS** plots for the Classification-based model: each of the 12 plots includes 3528 points associated with different choices of the agent parameters, all with the same initial opinion distribution and underlying digraph. Plots in the same row have the same initial opinion distribution (orange circle). Plots in the same column have the same underlying digraph (from left to right, Directed Ring, Scale-Free, Small-World, Complete). All the simulations were performed for 1000 time steps and 100 agents.

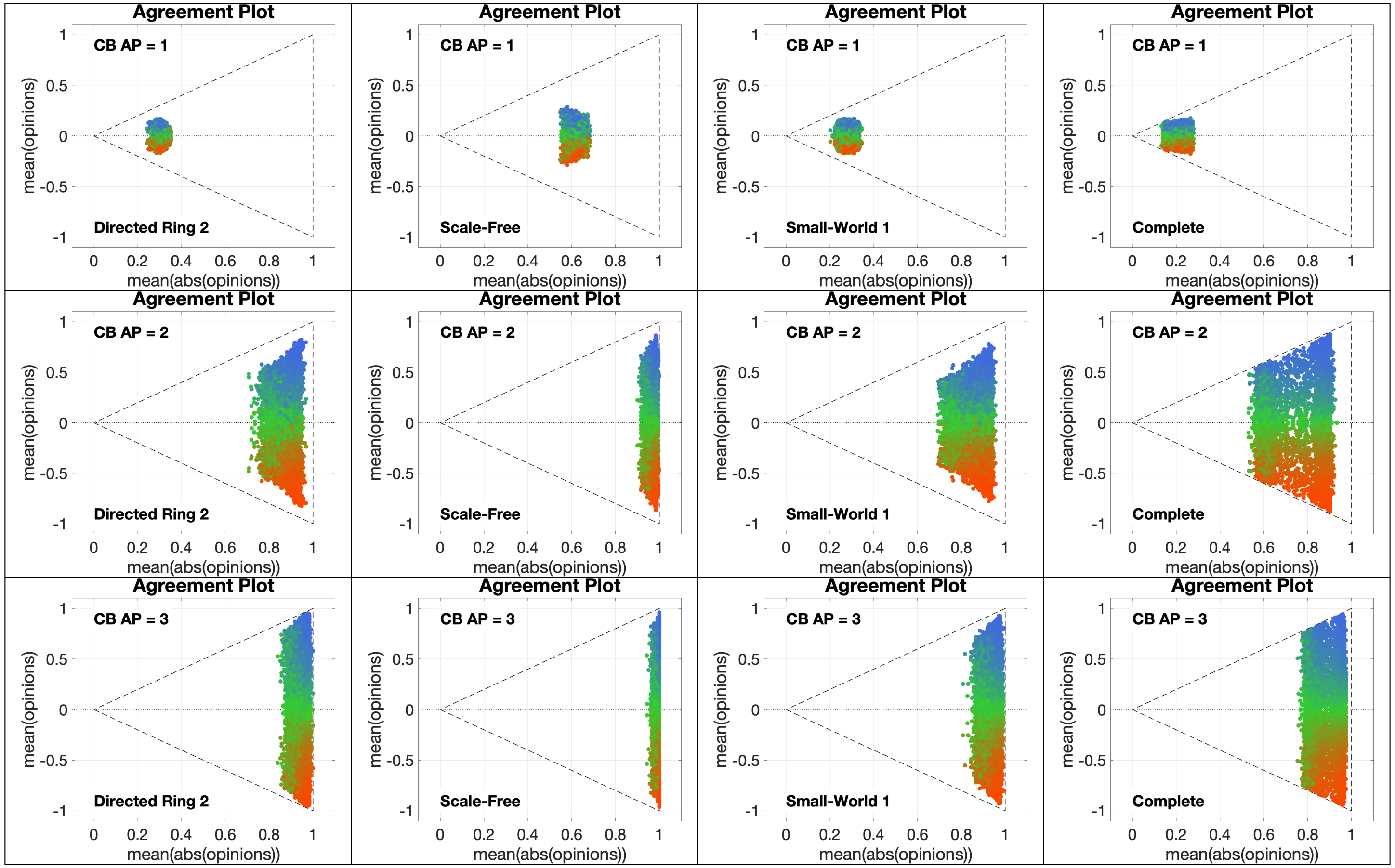

Table 18: **IOSS** plots for the Classification-based model: each of the 12 plots includes 5314 points associated with different choices of the initial opinion distributions, all with the same agent parameters and underlying digraph. Plots in the same row have the same agent parameters. Plots in the same column have the same underlying digraph (from left to right, Directed Ring, Scale-Free, Small-World, and Complete). All the simulations were performed for 1000 time steps and 100 agents.
